# Plant Interaction Patterns Shape the Soil Microbial Community and Nutrient Cycling in Different Intercropping Scenarios of Aromatic Plant Species

**DOI:** 10.3389/fmicb.2022.888789

**Published:** 2022-05-27

**Authors:** Yue Sun, Li Chen, Shiyi Zhang, Yantao Miao, Yan Zhang, Zhenglin Li, Jingya Zhao, Lu Yu, Jie Zhang, Xiaoxiao Qin, Yuncong Yao

**Affiliations:** ^1^Beijing Advanced Innovation Center for Tree Breeding by Molecular Design, Beijing University of Agriculture, Beijing, China; ^2^College of Plant Science and Technology, Beijing University of Agriculture, Beijing, China; ^3^Beijing Key Laboratory for Agricultural Application and New Technique, Beijing University of Agriculture, Beijing, China; ^4^College of Biological Sciences and Technology, Beijing Forestry University, Beijing, China

**Keywords:** aromatic plant intercropping, management pattern, C and N cycles, microbial PLFAs, diversities of microbial communities

## Abstract

Intercropping systems improve the soil nutrient cycle through microbial community activity and then land productivity. However, their interactions mechanism underlying that the mixed aromatic plant species intercropping regulate the soil microbiome and nutrient cycling on the perennial woody orchard is still uncovered. We designed treatments with 0, 1, and 3 aromatic plant species intercropped in two scenarios of clean tillage (T model, T1, T2, and T4) and natural grass (G model, G1, G2, and G4) in apple orchards, and investigated intercrops effects at the branch growing stage (BGS) and fruit development stage (FDS), respectively. Compared with T model, G model in FDS increased alpha diversity of bacterial community and Shannon index fungal community, the relative abundance of dominant taxa, such as Acidobacteria and Actinobacteria, and also the numbers of up and down-regulated OTUs, the most of indices of co-occurrence network in both bacterial and fungal community, and then improved invertase activity and available nitrogen content. Relative to G1, G2 and G4 reduced diversity bacterial community in FDS, the relative abundance of dominant taxa, the most of indices of co-occurrence network, and then improved soil invertase activity and total phosphorus content in soil. Moreover, Shannon index of fungal community, the altered number of OTUs and the most indices of co-occurrence network were higher in G4 than those in G2 in FDS. These changes above in FDS were more markedly than those in BGS, suggesting that chemical diversity of litter from mixed species of aromatic plants in natural grass scenario led to diversity, complexity, and stability of soil microbial community and then nutrient cycling. It provided a novel highlight and method to modulate biocenosis and then improve the soil nutrient cycling.

## Introduction

Intercropping has become a potential strategy for agroecological intensification through plant species interactions that introduce greater plant diversity and functional complementarity in crop systems (Bommarco et al., 2013), as it can strengthen the regulation and support of ecosystem multifunctions and services by affecting the efficiency of primary production, decomposition of organic matter and recycling of biologically essential nutrients (Cardinale et al., 2012; Bommarco et al., 2013). Many studies have shown that the interactions and functional complementarity between plant species in crop intercropping systems, grassland ecosystems and forestry ecosystems promote stocks of carbon (C) and nitrogen (N) and, thus, productivity in soil (Fornara and Tilman, 2008; Steinbeiss et al., 2008). However, the plant species interactions in compound agroforestry ecosystems, such as orchard ecosystem, are different from those in the aforementioned systems and ecosystems to some extent. One is because the orchard ecosystem includes tall perennial woody plants and low annual herbaceous plants, there is unequal competition between them, not only in light and humidity aboveground, but also in soil nutrients and water underground, resulting in the different ecosystem space and dynamic changes of nutrient cycling during fruit plant growth and development period (Gao et al., 2013). The other is because in the agroforestry ecosystem, annual rotation and clean tillage is not required in tall perennial woody plants, leading to the more increasing of soil organic matter and abundance of beneficial microbial community than that crop system. And finally, there is different in growth characters, product form and management practices, due to perennial fruit plants usually remain in production for 20 years or more (Castellano-Hinojosa and Strauss, 2020). These suggest more complex mechanisms of the soil microbial community associated with mediating nutrient cycling in these systems. Moreover, planting multispecies mixtures, such as the combination of at least two legume or non-legume species, may provide additional benefits by not only increasing microbial diversity but also the abundance of beneficial soil microbes compared with monocultures (Wortman et al., 2012). However, how mixed intercropping with aromatic plants regulate nutrient cycling by modulating the diversity, composition, and co-occurrence network of microbial community is still unclear.

Soil microbes are strongly associated with several ecosystem processes, including plant litter decomposition, organic matter degradation and C and N cycling, through interactions with plants in the soil (Bulgarelli et al., 2012; Ye et al., 2019). These processes are inevitably impacted by local biotic and abiotic conditions (van der Putten et al., 2013), the local vegetation patterns and their intraspecies and interspecies interactions, as well as the introduction of new plant species, which destabilize the microbial communities and their function in the rhizosphere and soil bulk (Bardgett and Wardle, 2003) due to changes in vegetation composition and plant litter composition and decomposition (Cong et al., 2015). When undertaking soil decomposition and mediating C and N biogeochemical cycles, soil microbes have the ability to adapt to the composition of different resources and can thereby alter their nutrient use efficiencies (Whipps, 2001; Cardon, 2004; Falkowski et al., 2008; Wagg et al., 2014; Bever, 2015). For example, when a new plant is introduced or invaded, the dynamic changes in soil microbes lead to a corresponding change in soil C accumulation (Scherer Lorenzen and Potvin, 2007; Mooshammer et al., 2014; Zechmeisterboltenstern et al., 2015; Zhou et al., 2017). A hump-shaped relationship between the soil net C accumulation rate and rhizosphere microbial biomass in intercrop ecosystems showed that soil C accumulation may be either enhanced or reduced depending on microbe abundance (Ni et al., 2020). In addition, supplementing stands with leguminous plants promoted the growth and diversity of soil microbial communities and then increased the soil C content in Chinese fir plantations due to the high-N litters of leguminous plants (Oli et al., 2018; Zhang et al., 2020a,b). Moreover, previous studies have proposed the introduction of functional plants into plantations as a way of improving the ecosystem of the understorey and the diversity of the microbial community, thus improving the soil ecosystem (Briones and Ineson, 1996; Mctiernan et al., 1997; Ge et al., 2011; Sun et al., 2018; Zhang et al., 2020a), which, *via* the diverse soil environment created by plant vegetation and litter to improves the diversity of the microbial community and then ensure sufficient nutrient availability (Cardon, 2004; Cleveland and Liptzin, 2007; Spohn, 2016; Li et al., 2017). It follows that the soil microbial communities are influenced by the interactions between plants and microbes, which are affected by any changes in soil conditions following vegetation changes and plant litter diversity (Zhang et al., 2011; Spohn, 2016; Ren et al., 2018). However, a clear understanding of the effects of introducing new plant species in different vegetation conditions and how synergistic symbiosis and antagonism in plant-plant and plant-microbial interactions alter stable properties in microbial communities mediated nutrient cycling in woody-herbaceous co-plantation is still lacking.

Nutrient cycling plays essential roles in agroforestry soil ecosystems because it is directly actuated by soil microbial communities and positively linked to the supply of nutrients to vegetation (Weil and Magdoff, 2004). When intercropping is conducted with two or more species, the aboveground plant can enhance different litter inputs into the soil and, hence, promote the build-up of organic matter and C sequestration *via* microbial community interactions (Ghosh, 2006; Yang et al., 2010; Li et al., 2011). Recent studies have shown that there is a greater input of C into the soil through root residues in intercropping systems than in monocultures (Li et al., 2005). N storage is enhanced through the promotion of biological N fixation by legumes when intercropped with cereals, and improved N capture in mixed crops occurs through complementarity in foraging space (soil profile) and time (crop growth period) (Li et al., 2009). This is one of the main reasons why intercropping systems may facilitate greater crop N utilization, resulting in higher yields, than single-crop systems (Li et al., 2009). These results show that the sequestration of C and N and enhancement of productivity are attributed to the increase in plant species and litter diversity, which improve soil microbial communities in intercropping systems (Li et al., 2009; Lithourgidis et al., 2011; Santonja et al., 2017; Ye et al., 2019). However, litter diversity was proven to enhance organic matter decomposition by microbial communities, which could prevent the accumulation of C in the soil despite greater inputs. In addition, high N inputs in agroforestry ecosystems can suppress biological N fixation by legumes, thereby potentially reducing complementarity effects (Cardinale et al., 2012; Hooper et al., 2012). These results suggest that the interactions of plant species and environmental factors have more complex effects on the composition, structure, diversity, and stability of the soil microbial community in the compound agroforestry ecosystem.

Aromatic plants are a source of essential oils, cosmetics, and biocides (Lubbe and Verpoorte, 2011; Tang et al., 2013; Song et al., 2014). In previous studies we selected some species of aromatic plant that adapted for orchard according to the plant size, resistance of shade and essential oil composition. Some previous studies have shown that intercropping aromatic plants in orchards not only plays a positive role in improving soil nutrient status, inhibiting harmful pests and pathogenic fungi and enhancing soil microbial community diversity and stability, but also in enhancing the additive income of farmers from aromatic plant production (Song et al., 2013; Zhang et al., 2021). In addition, we found that various influences of different treatments with single species of aromatic plant intercrop on the arthropods community, soil microbial community, nutrient cycling and plant growth were different in orchard, for example, basil, summer savory and catnip (Labiatae) promoted the Shannon and Simpson indices of arthropod community while cornflower and ageratum (Compositae) reduced those in orchard (Song et al., 2010), and basil and summer savory (Labiatae) increased diversity of microbial community while ageratum (Compositae) decreased those (Zhang et al., 2021). These suggest that there will be different mechanism that intercropping with single species and with mixed species regulate soil microbial community in agroecosystem.

In the present study, we chose four kinds of aromatic plants Labiatae (basil and mint) and Compositae (french marigold and ageratum) as experimental materials, and designed treatments with 0, 1, and 3 plant species in two scenarios of clean tillage and natural grass. Aim is focus on identifying the effect on the mixed intercropping model with natural grass on soil microbiome and nutrient cycling in orchard system. It is considering the realistic status of apple cultivation in north China, where ground management employ natural grass to avoid the needless input by row weeding (traditional mode). Importantly, we hypothesis that the diversity of vegetation could regulates diversity and stability of soil microbial community.

## Materials and Methods

### Study Site Description

The field experiment was conducted during 2014–2016 in an apple orchard located in Changping District (115°50′E, 40°23′N) of Beijing, China. This study area has a typical temperate semihumid continental monsoon climate. The average annual rainfall is ~550 mm, and the average annual temperature is 11.6°C (meteorological data recorded by weather stations in Changping District). The soil type in the orchards is sandy loam, “Fuji” (Malus domestica cv. Fuji/M. prunifolia (Willd).) apple trees were planted at a row spacing of 3 × 6 m in 1999. The test orchard has been managed using organic farming practices since 2005 and was certified as an organic farm in 2007.

### Experimental Design

The experimental site in an apple orchard was divided into the clean tillage area and the natural grass area in each row in 2007. In the clean tillage area, all native vegetation, weed roots and grass were artificially regularly removed each year. Then, the cultivation mode of clean tillage (T model) and intercropping with 0, 1, or 3 species of aromatic plants was constructed (T1, T2, and T4). In the natural grass (included 14 species, Supplementary Figure S1) area, the native vegetation was retained under 25 cm with mechanical mowing (Song et al., 2013). Then, the cultivation of natural grass (G model) and intercropping with 0, 1, or 3 species of aromatic plants were performed (G1, G2, and G4). There were 18 plots in total, which each plot was 30 m long and 5 m wide and included 20 apple trees and 7 strips aromatic plants, with three plots in each treatment. The different treatments were separated through a 10 m isolation belt, and different pattern were separated by a 20 m isolation belt (Supplementary Figure S1). In previous study, we found that intercropping with Basil, French marigold, Mint, and Ageratum significantly regulated arthropods community and microbial community, while these effects were different in orchard (Song et al., 2010; Zhang et al., 2021). Therefore, we chose these four kinds of aromatic plants as experimental materials for study. The plant species in natural grass scenario were identified by the botanist in Department of Botany, Beijing Agricultural University. The single-species intercropping only included basil (*Ocimum basilicum* L.), and the mixed-species intercropping included the mixed french marigold (*Tagetes patula* L.), mint (*Menta haplocalyx* Briq.), and ageratum (*Ageratum houstonianum* Mill.). CK plots (0 species) were arranged without intercropped aromatic plants in the two models. The single aromatic plant species was intercropped with basil in 7 strips, and the 3 mixed species were intercropped with French marigold in 2 strips, mint in 3 strips and ageratum in 2 strips. Seeds of the intercrops were sown in a greenhouse every January, and seedlings were transplanted in mid-March to the apple orchard at a spacing of 0.2 × 0.3 m, 0.5 m away from the apple tree trunks, when they were ~10 cm tall. The aromatic plants in the intercropped plots were mowed in late September annually, with the plant residues left *in situ*.

The intercropping plots were mowed by regular mechanical mowing and retained vegetation at 25 cm tall after mid-April, leaving plant residues to decompose *in situ*. All treatments were arranged in a random block design with three replicates.

### Soil Sampling

Soil samples were collected from the T and G models at the BGS (mid-May) and FDS (mid-August) of the apple trees from 2014 to 2016. The surface soil was removed, and soil samples were collected from the 0 to 40 cm layer using a stainless-steel corer (4.5 cm inner diameter) at 1–2.5 m from 6 randomly selected tree trunks in each plot. Fresh samples (total 3.0 kg) from six locations were randomly collected and pooled together as one sample, and then sieved (<2 mm) and divided into three composite samples per plot. One sample was immediately stored at −80°C for DNA extraction, the second was stored at 4°C to test the soil water content (SWC) and enzymatic activity, and the third was air dried and stored at room temperature before soil chemical analysis. All soil chemical properties were measured and analyzed within 2 weeks at the Beijing Key Laboratory for Agricultural Applications and New Techniques of Beijing University of Agriculture, Beijing.

### Soil Chemical and Enzyme Activity Analyses

Soil pH was measured in a mixture of soil and water (1:2.5) according to Chaturvedi et al. (2006), and the soil water content (SWC) was determined gravimetrically by drying at 105°C. Total soil organic content (SOC) was measured by dichromate oxidation (Hirota et al., 2005; Anderson Teixeira et al., 2010). Total nitrogen (TN) was measured by the Kjeldahl method (Hirota et al., 2005), and the available nitrogen (AN) was extracted using the microdiffusion method (Hirota et al., 2005). Soil total phosphorus (TP) was determined using a flame photometer (Borie et al., 1989), while available P (AP) was extracted by the Olsen method (Li et al., 2004). Soil urease (URE) and invertase (INV) activities were determined by spectrophotometry at 578 and 508 nm, respectively (Wang et al., 2009). The soil acid phosphatase (ACP) activity was determined as fluorescence (van Aarle and Plassard, 2010).

To obtain a quantitative ecosystem multifunctionality index for the soils (SMF) of each site, we selected the nutrient properties related to C and N cycling to construct a soil multinutrient cycling index similar to the widely used multifunctionality index. These nutrient properties deliver fundamental support and regulatory ecosystem services. The individual functions were subjected to Z score transformation (Delgado Baquerizo et al., 2016). The standardized rates of ecosystem functions were then averaged to obtain a multifunctionality index (Delgado Baquerizo et al., 2016). SMF is composed of the three enzyme activities mentioned above and is related to the cycling of organic matter in soil ecosystems.

### Soil Phospholipid Fatty Acid (PLFA) Analysis

The extraction and analysis of phospholipid fatty acids (PLFAs) were performed according to the FAME method. The procedure was as follows: 15 ml of 0.2 mol/L KOH methanol solution and 10 g of fresh soil sample were added to a 50 ml centrifuge tube, mixed evenly, and incubated at 37°C for 1 h (for release of phospholipid fatty acids and esterification). Then, 3 ml of 1 mol/L acetic acid solution was added to neutralize the pH, and the mixture was shaken well. Next, 10 ml of n-hexane was added, and the FAMEs were transferred to the organic phase. The solution was centrifuged at 3,500 r/min for 10 min, and the upper n-hexane was transferred to a clean test tube, while the solvent was volatilized under N2 gas flow. PLFAs were dissolved in 0.5 ml of 1:1 (V/V) n-hexane (methyl-tertbutylether) for GC analysis. Using an Agilent Technologies 6890N Network QC System and Agilent 5973 Network Mass Selective Detector, the standard sample and PLFAs mixture to be tested were analyzed in parallel under the chromatographic conditions described by Zhang et al. (2008). The identification of PLFAs were based on the Sherlock MIS 4.5 system (Sherlock Microbial Identification System, MIDI, Newark, Delaware, USA).

### Soil DNA Extraction and Sequencing

To investigate the soil microbial community, genomic DNA from four soil samples was extracted from 0.25 g samples using a TIANamp Soil DNA Kit (Tiangen Biotech, Beijing, China), which was used as a template in the following PCR amplicon. The primers 341F and 785R (Klindworth et al., 2013) and ITS regions (ITS3_KYO2-F and ITS4_KYO3-R) (Toju et al., 2012) were used to amplify the V3–V4 hypervariable regions of bacterial 16S rRNA and fungal ITS3-ITS4 hypervariable regions of fungi, respectively. The purified PCR amplicons were sequenced by the Illumina MiSeq (300 bp paired-end reads) platform from Ori-Gene Technology Co., Ltd. (Beijing, China). Then, the 16S and ITS sequences of the high-quality paired-end reads were merged by FLASH software (Magoc and Salzberg, 2011), and the barcodes of the final sequences were filtered and removed by Mothur (https://mothur.org). The operational taxonomic units (OTUs) were clustered at 97% similarity based on the merged sequences using the UPARSE pipeline (Edgar, 2013). The OTUs were generated and aligned against by the SILVA and UNITE databases and the RDP classifier (https://rdp.cme.msu.edu/) for the 16S and ITS sequences to obtain the taxonomic information, respectively (Pruesse et al., 2007). Moreover, alpha diversity indices (Sob, Chao, and Shannon) were calculated with Mothur v. 1.34.4 (Schloss et al., 2009).

The Sob (observed richness), Chao1 (non-parametric estimator of richness), and Shannon (non-parametric Shannon index) indices of the microbial community were calculated to estimate the alpha diversity of each sample (Campbell et al., 2013). Linear regression analysis was applied to test the correlation of aromatic plant species richness diversity of microbial communities. The structure of the microbial community (as determined by both genomes) was analyzed by principal component analysis (PCA) with the relative abundances of the dominant populations at the phylum, class, order, and genus levels. To demonstrate the relationship of different microbes among several treatments, co-occurrence network analysis based on Spearman's rank analysis was performed using the 50 most abundant genera of bacterial and fungal communities. The co-occurrence patterns of soil microbial communities were explored based on strong (ρ > 0.7) and most significant correlations (*P* < 0.001) and were visualized with Gephi (Version 0.9.2). The size of each network node represents the number of connections, the node is colored by taxonomy, and the edge is colored by correlation type (positive or negative). Spearman's rank analysis of bacterial and fungal abundances was performed at the 0.05 probability level in R software (Version 4.0.2) (Jacomy et al., 2009).

### Statistical Analysis

Differences among treatments were analyzed by analysis of variance (ANOVA), where parameter assumptions of normality were determined using Duncan's multiple range test. All statistical analyses were carried out using R software. Differences were not significant at *P* > 0.05 but were significant at *P* ≤ 0.05 and *P* ≤ 0.01.

## Results

### PLFA Changes in the Soil Microbial Community

Compared with T1, T2 reduced the total, AMF and G^−^ biomass, leading to a high G^+^/G^−^ ratio in the BGS, and enhanced all PLFAs, leading to a low B/F ratio in the FDS. T4 enhanced all PLFAs, leading to a high G^+^/G^−^ ratio in the BGS, and enhanced the total, fungal and G^+^ biomass but reduced G^−^ biomass, resulting in a high G^+^/G^−^ ratio and low B/F ratio in the FDS. Differences in T4 relative to T2 regarding the increase in the BGS and decrease in the FDS associated with the PLFAs effects were noted.

**Figure 1:**
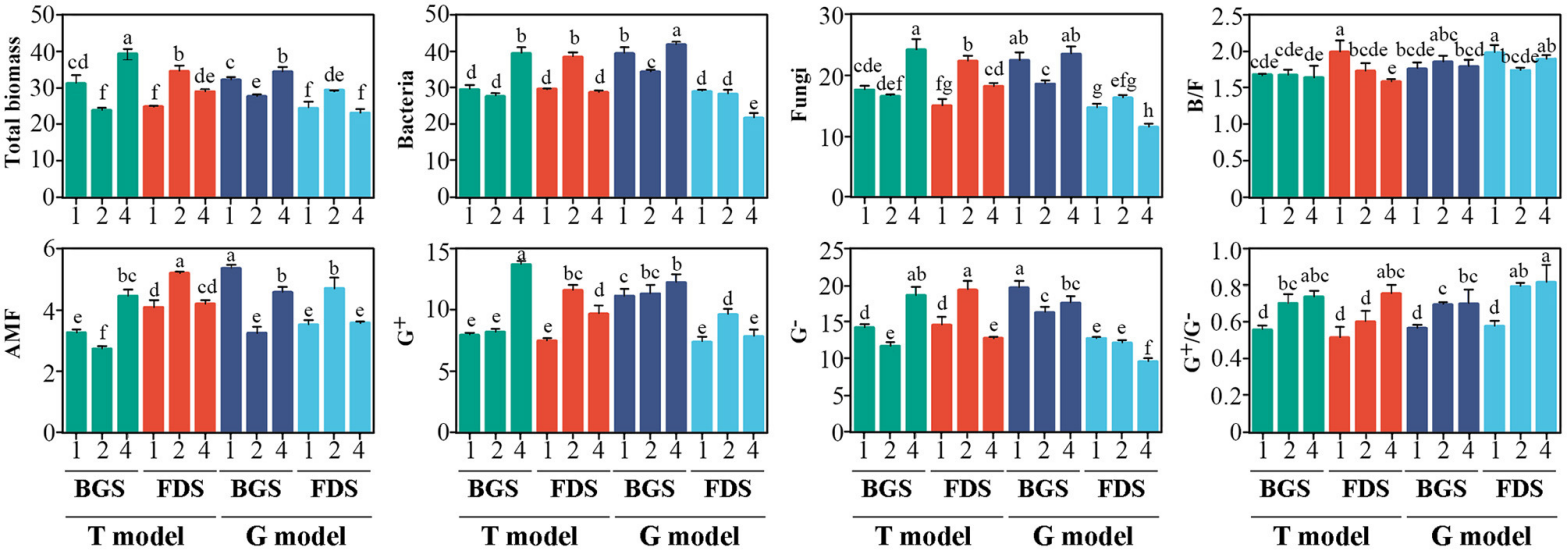
Variation in soil microbial PLFAs between the intercropping patterns and between the development stages in the studied soils. B/F, bacterial biomass/fungal biomass ratio; AMF, arbuscular mycorrhizal fungi; G^+^, gram-positive bacteria; G^−^, gram-negative bacteria; G^+^/G^−^, gram-positive bacteria/gram-negative bacteria ratio. 1, 2, and 4 indicate intercropping with 0, 1, and 3 species of aromatic plants, respectively, to facilitate regression analysis. BGS, branch growth stage; FDS, fruit development stage. T model, intercropping with aromatic plants in the clean tillage soil; G model, intercropping with aromatic plants in the natural grass soil. Different letters indicate significant differences at *P* < 0.05 based on Duncan's multiple range test.

Relative to G1, G2 reduced the total, bacterial, fungal, AMF, and G^−^ biomass in the BGS but enhanced the total, AMF, and G^+^ biomass in the FDS, resulting in a high G^+^/G^−^ ratio in the BGS and FDS and a low B/F ratio in the FDS. G4 enhanced the biomass of total, bacteria and G^+^ in the BGS but reduced the biomass of bacteria, fungi, and G^−^ in the FDS, with the G^+^/G^−^ ratio being higher than that in G1 but not higher than that in G2 soil. These change trends in the G model were similar to those in the T model, while the effects of both G2 and G4 on some PLFAs, as well as their differences, were different, especially the effects of G4 on the bacterial, fungal, and G^−^ biomasses, which were lower than those of G1 in the FDS, although there were no significant differences in the B/F and G^+^/G^−^ ratios between G4 and G2 (Figure 1).

The effects of the developmental stages of the host and intercrops on the PLFAs were generally higher than those in the models, and the differences in the BGS and FDS in the G model were greater than those in the T model, especially in the FDS, where G4 relative to G2 significantly reduced bacterial, fungal, and G^−^ biomass, which was different from T4. Correspondingly, G2 reduced most of the PLFA indices in the BGS and enhanced them in the FDS, similar to T2 and contrary to G4. These results suggested that the developmental stage and intercropping patterns had significant coregulatory effects on PLFAs.

### The Taxon Structure and OTU Proportion of the Microbial Community

Across all the samples, we obtained a total of 2,102,831 and 2,213,651 high-quality bacterial and fungal sequences, which were grouped into 16,991 and 3,267 OTUs, respectively, when using the 97% sequence similarity cut-off. The composition of the total bacterial community was dominated by the following phyla: Proteobacteria (up to 44.19%), Acidobacteria (up to 19.51%), Bacteroidetes (up to 12.02%), Actinobacteria (up to 9.98%), Planctomycetes (9.10%), Gemmatimonadetes (up to 5.51%), and Verrucomicrobia (up to 5.99%). In addition, Nitrospirae had a relative abundance of 1.24%. The composition of the total fungal community was dominated by the following phyla: Ascomycota (up to 44.37%), Basidiomycota (up to 44.06%), and Zygomycota (up to 39.88%) (Supplementary Tables S1,S2).

Compared with T1, T4 decreased the percentages of all bacterial taxonomy at the phylum level in the BGS, while both T2 and T4 decreased those at the class, order, family, and genus levels in the FDS. However, G2 and G4 promoted that percentage at the class, order, family, and genus levels in the BGS and decreased the percentage at the phylum and class levels in the FDS, with no differences between G2 and G4.

**Figure 2:**
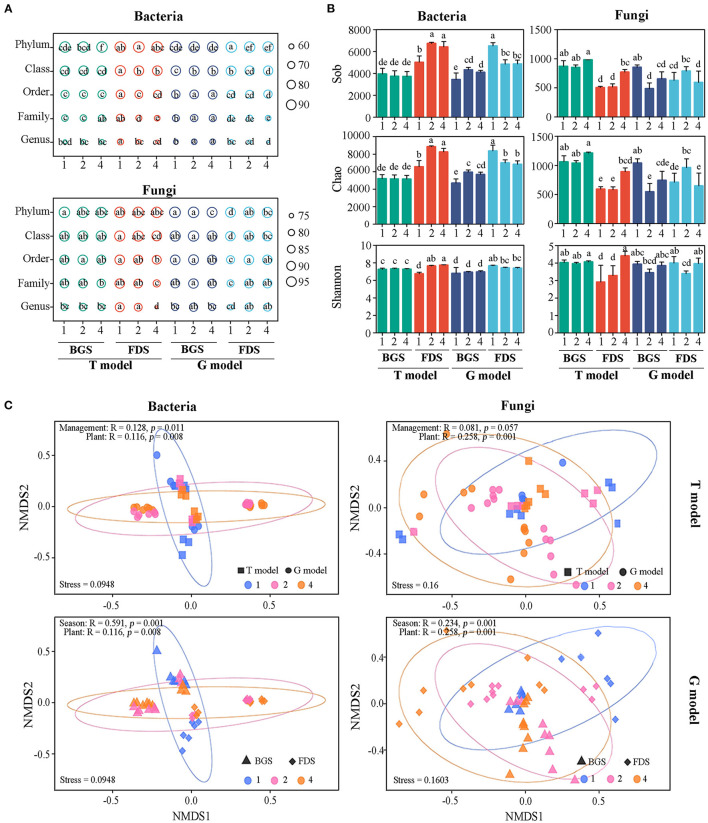
Variation in the structure, alpha diversity and beta diversity of soil microbial communities between the intercropping patterns and the development stages. **(A)** The percentage of the total microbial community at the taxon level for the bacterial and fungal communities. **(B)** Alpha diversity of bacterial and fungal communities. Sob was the observed operational taxonomic units (OTUs); Chao was used to evaluate community richness based on OTUs; and the Shannon index was used to assess community diversity. **(C)** Beta diversity of bacterial and fungal communities. NMDS shows the structures of the soil bacterial and fungal communities. Ninety-five percent confidence ellipses are shown around the samples grouped based on intercropping soil. Similarity values, which are shown in each plot, among the samples of plant species richness of IAP between the intercropping patterns and development stages were examined *via* ANOSIM. 1, 2, and 4 indicate intercropping with 0, 1, and 3 species of aromatic plants, respectively, to facilitate regression analysis. BGS, branch growth stage; FDS, fruit development stage. T model, intercropping with aromatic plants in the clean tillage soil; G model, intercropping with aromatic plants in the natural grass soil. Different letters indicate significant differences at *P* < 0.05 based on Duncan's multiple range test.

Similarly, only T4 decreased the percentage of all fungal taxonomies at the class, order, family, and genus levels in the FDS, with a value less than that for T2, while both G4 and G2 increased the percentage at all taxon levels, with no differences between G2 and G4. G4 decreased only the percentage at the phyla level, with a value less than G2 (Figure 2A).

The percentage of taxa (phylum, class, order, family, and genus) relative to the total bacterial community in the BGS was lower than that in the FDS in the T model, contrary to that in the G model. The percentage of taxa relative to the total fungal community in T4 in the BGS was higher than that in the FDS in the T model, while the percentages of G4 and G2 in the BGS were relatively lower than those in the FDS in the G model due to the lower percentage of G1 in the FDS.

### The Alpha Diversity of the Microbial Community

Compared with T1, T2, and T4 promoted the Sob, Chao, and Shannon indices of the bacterial community in the FDS but not in the BGS. G2 and G4 significantly increased Sob and Chao indices of the bacterial community in the BGS but decreased them in the FDS relative to those in G1. There were fewer differences between the single species and the mixed species of IAP than between the two development stages (Figure 2B). The regression analysis indicated that the alpha diversity (Sob, Chao, and Shannon indices) of the bacterial community displayed a linear decrease (no significant difference) in the G model, in contrast to the T model, as aromatic plant species richness increased (Supplementary Figure S2). These results suggested that the effects of IAP on alpha diversity according to the T model were reduced by the G model, with significant functional complements of development stages.

T4 significantly promoted the alpha diversity of the fungal community compared with T1 in FDS. However, compared with G1, both G2 and G4 decreased Sob and Chao of fungal community in the BGS, and G2 significantly promoted the Chao index in the FDS. G2 also decreased the Shannon index in the BGS and FDS (Figure 2B). The regression analysis indicated that the Shannon index of the fungal community displayed a linear increase, while the Sob and Chao indices showed a linear decrease in the G model with the increase in aromatic plant richness (Supplementary Figure S2). These results suggested that the alpha diversity of the fungal community was associated with the developmental stages of larger plant biomass with less plant species richness and was linked to interactions among plant species with more plant species richness.

### The Beta Diversity of the Microbial Community

Non-metric multidimensional scaling (NMDS) analysis revealed that the soil samples formed distinct clusters in the ordination space depending on aromatic plant species richness between the two models and between the BGS and FDS (Figure 2C), with significant differences found at the taxonomic level (ANOSIM test). These differences among soils under different levels of aromatic plant species richness were larger for bacterial communities than for fungal communities, while differences between single and mixed beta-diversity were more distinct in the fungal community than in the bacterial community, which indicates that soil bacterial communities were possibly more influenced by models and development stages than fungal communities.

As aromatic plant species richness increased, the bacterial community similarity tended to increase in the T models, which was not significantly reduced in the G model, similar to the fungal community similarity. Bacterial community similarity tended to increase (not significantly) in the two developmental stages but was lower in the BGS than in the FDS, while fungal community similarity displayed a linear rise in the BGS and a decrease in the FDS, although these changes were not significantly different (Supplementary Figure S3). These results suggested that weak changes in beta diversity by the T model could be regulated by the G model, with few seasonal differences.

### The Composition of the Microbial Community

**Figure 3:**
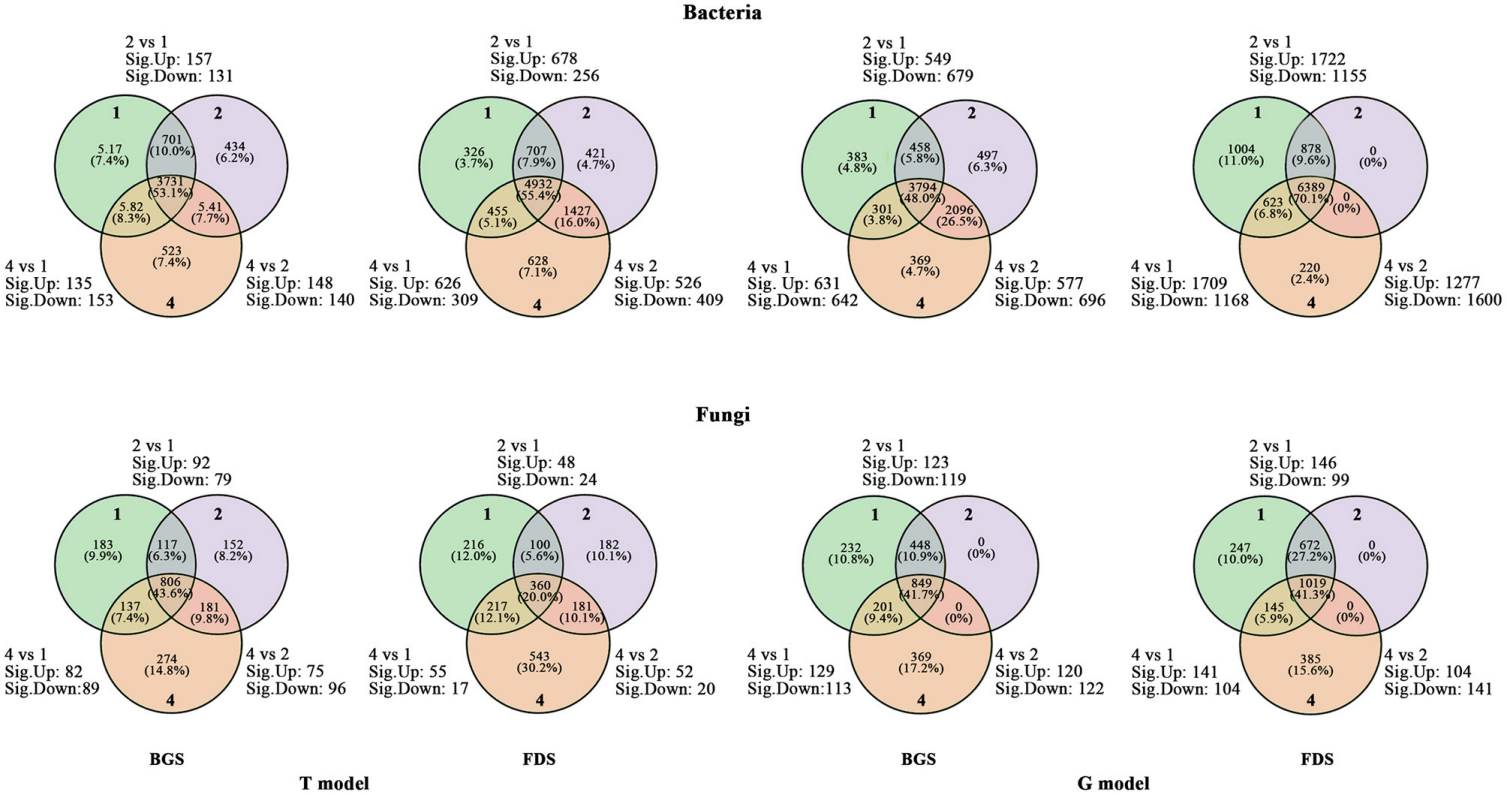
Venn diagram of regulated numbers of core OTUs between the intercropping patterns and between the development stages. 2 vs. 1, 4 vs. 1 and 4 vs. 2 indicate the comparison of different species intercropping for core OTUs, respectively. Sig. up and sig. down indicate the numbers of significantly upregulated core OTUs and significantly downregulated core OTUs, respectively. 1, 2, and 4 indicate intercropping with 0, 1, and 3 species of aromatic plants, respectively, to facilitate regression analysis. BGS, branch growth stage; FDS, fruit development stage. T model, intercropping with aromatic plants in the clean tillage soil; G model, intercropping with aromatic plants in the natural grass soil.

The upregulated numbers of core OTUs in the two communities were greater in T2 vs. T1 than in T4 vs. T1, and the downregulated numbers were lower in T2 vs. T1 than in T4 vs. T1, except for the fungal community in the FDS. The up- and downregulated numbers of core OTUs in the two communities in T4 vs. T2 were less than those in T2 vs. T1 and in T4 vs. T1 or were located between them. Both the up- and downregulated numbers of soil core OTUs of the bacterial community in the BGS were less than those in the FDS, and those of the fungal community were the opposite. The numbers of upregulated core OTUs in the two communities were lower in G2 vs. G1 than in G4 vs. G1 in the BGS but higher in the FDS, and the numbers of downregulated OTUs were higher in G2 vs. G1 than in G4 vs. G1 in the BGS but lower in the FDS. The numbers of upregulated core OTUs in the two communities were the lowest, and the numbers of downregulated OTUs were the highest in G4 vs. G2, relative to G2 vs. G1 and G4 vs. G1. The numbers of both up- and downregulated OTUs in the bacterial community in the BGS were also lower than those in the FDS, the numbers of upregulated OTUs in the fungal community in G2 vs. G1 and in G4 vs. G1 were lower, those in G4 vs. G2 were higher, those in BGS were higher than those in FDS, the numbers of downregulated OTUs were higher, and those in G4 vs. G2 were lower in the BGS than in the FDS (Figure 3). This result suggested that the soil core taxa of the two communities were possibly activated by single and mixed intercropping aromatic plants in different ways.

**Figure 4:**
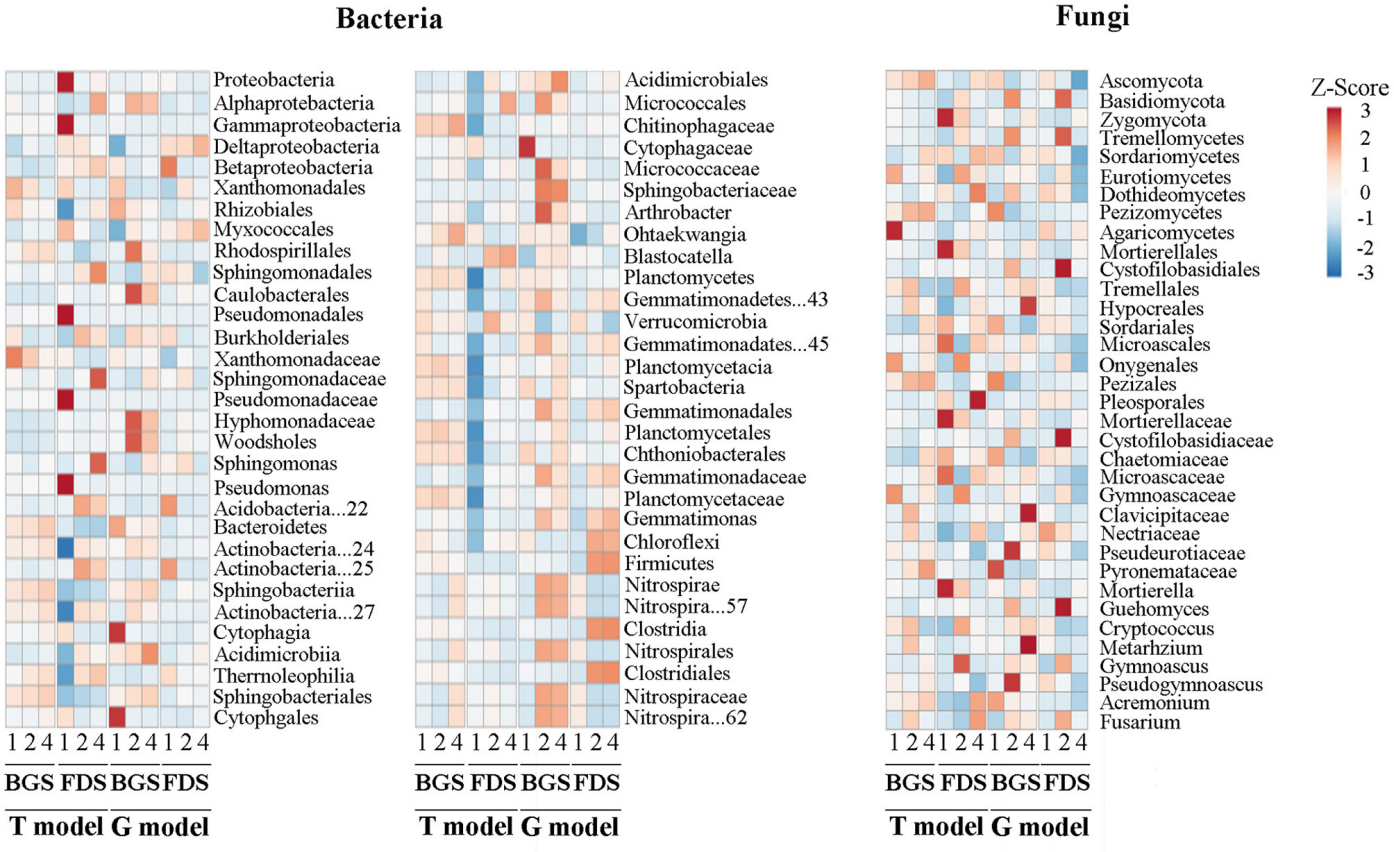
The variation in the composition and structure of the microbial community at the taxon level between the intercropping patterns and between the development stages in the studied soils. 1, 2, and 4 indicate intercropping with 0, 1, and 3 species of aromatic plants, respectively, to facilitate regression analysis. BGS, branch growth stage; FDS, fruit development stage. T model, intercropping with aromatic plants in the clean tillage soil; G model, intercropping with aromatic plants in the natural grass soil.

Compared with the respective CK, IAP in the T model altered the relative abundance of a few bacterial taxa in the BGS, while it upregulated the relative abundance of almost all dominant bacteria and their taxonomic members and downregulated the abundance of Proteobacteria, Gammaproteobacteria included Pseudomonadales in the FDS. IAP in the G model upregulated the abundance of Proteobacteria, including Alpha- and Delta- and Beta-proteobacteria, Acidobacteria, Actinobacteria, Gemmatimonadetes, Planctomycetales, and Nitrospirae, as well as most of their taxonomic members, and downregulated the abundance of Bacteroidetes, including Cytophagia and Verrucomicrobia (e.g., Spartobacteria, Chloroflexi, and Firmicutes), in the two development stages. Compared with T2, T4 upregulated the abundance of Sphingobacteriaceae, Nitrospirae and its taxonomic members in the BGS and upregulated the abundance of Proteobacteria, Alphaproteobacteria and its genus, Micrococcales and Planctomycetes and downregulated the abundance of Acidobacteria and Verrucomicrobia in the FDS. Similarly, G4 upregulated the abundance of Acidimicroiia, Acidimicrobiales, and Spartobacteria, downregulated the abundance of Caulobacterales and Micrococcales, as well as their family and genus members in the BGS, and downregulated the abundance of Sphingomonadales, Sphingomonadaceae, Sphingomonas, and Blastocatella, in the FDS (Figure 4).

Compared with the respective CK, T2 in the BGS significantly upregulated the relative abundance of 4 members in Ascomycota and downregulated that of the other 3 members in Ascomycota and 1 member in Basidiomycota, T4 upregulated the abundance of 3 members in Ascomycota and downregulated that of 3 members in Basidiomycota, in which 3 members in Basidiomycota and 1 member in Ascomycota were upregulated, and 2 members in Basidiomycota and 1 member in Ascomycota were downregulated, in T4 relative to T2. T4 in the FDS upregulated the abundance of Basidiomycota and its 2 members and 4 members in Ascomycota and downregulated that of the other 4 members in Ascomycota. T4 upregulated the abundance of 6 members in Ascomycota and downregulated that of Zygomycota and its members, whereby Ascomycota and its 8 members were upregulated and the other 3 members in Ascomycota, Basidiomycota, and Tremellomycetes were downregulated in abundance relative to T2. G2 in the BGS upregulated the abundance of 4 members in Basidiomycota and 5 members in Ascomycota and downregulated that of Ascomycota and 6 other members. G4 upregulated the abundance of 4 members in Ascomycota and downregulated that of Ascomycota and 4 other members in Tremellomycetes, whereby 3 members in Ascomycota were upregulated and 4 members in Basidiomycota were downregulated in abundance relative to G2. G2 in the FDS upregulated the abundance of Basidiomycota and 4 members and 5 members in Ascomycota and downregulated that of the other 2 members in Basidiomycota and Ascomycota. G4 downregulated that of Ascomycota and 8 members, with Basidiomycota and 4 members and 5 members in Ascomycota being downregulated in abundance relative to G2 (Figure 4).

### Co-occurrence Network of the Microbial Community

**Figure 5:**
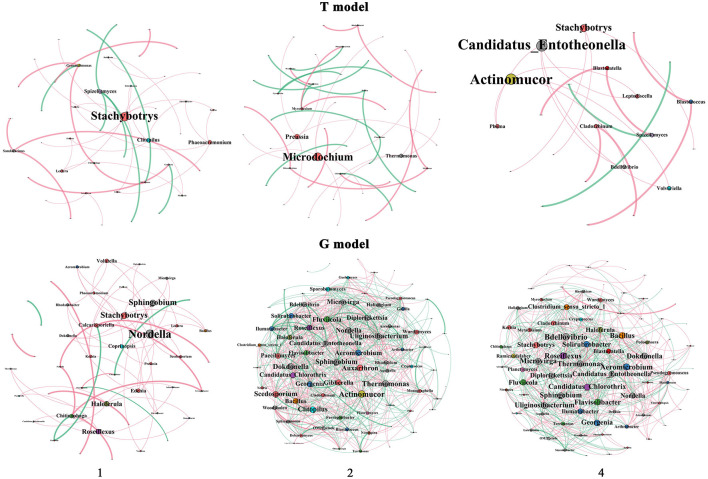
Coccurrence network of the microbial community between the intercropping patterns. 1, 2, and 4 indicate intercropping with 0, 1, and 3 species of aromatic plants, respectively, to facilitate regression analysis. T model, intercropping with aromatic plants in the clean tillage soil; G model, intercropping with aromatic plants in the natural grass soil.

From the properties of the co-occurrence network of both the bacterial and fungal communities, the levels of most network indices were reduced by T2 and T4 relative to T1 in the T model, except N (nodes), NE (negative edges), MD (modularity), and NC (number of communities), in which all indices of the network except AD (average degree) and D (density) in T4 were lower than those in T2. However, the levels of most network indices were enhanced by G2 and G4 relative to G1 in the G model, in which E (edges), PE (positive edges), NE, AD, ACC (average clustering coefficient) and D in G4 were lower than those in G2 (Figure 5, Supplementary Table S3).

There were 4, 3, and 2 hubs (>4 degrees) in T1, T2, and T4 in the T model soil, respectively, mainly including Stachybotrys and Phaeoacremonium (Ascomycota), Spizellomyces (Chytridiomycota), and Clitopilus (Basidiomycota) in T1, Microdochium and Preussia (Ascomycota) and Thermomonas (Proteobacteria) in T2, and Candidatus Entotheonella (Proteobacteria) and Actinomucor (Zygomycota) in T4. However, there were 10, 63, and 62 hubs (>4 degrees) in G1, G2, and G4, respectively, mainly including 2 (Nordella and Sphingobium) members of Proteobacteria, Roseiflexus of Chloroflexi, Haloferula of Verrucomicrobia, Chitinophaga of Bacteroidetes, and Coprinopsis of Basidiomycota and 4 (Stachybotrys, Calcarisporiella, Edenia, and Volutella) members of Ascomycota in G1; 19 members of Proteobacteria, 16 members of Ascomycota, 6 members of Basidiomycota, 7 members of Actinobacteria, 5 members of Bacteroidetes, 2 members each of Chloroflexi, Firmicutes, and Planctomycetes, and 1 member each of Zygomycota, Verrucomicrobia, Nitrospirae, and Acidobacteria in G2; and 20 members of Proteobacteria, 17 members of Ascomycota, 6 members of Actinobacteria, 4 members of Bacteroidetes, 3 members of Basidiomycota, 2 members each of Chloroflexi, Firmicutes, Verrucomicrobia, Acidobacteria, and Zygomycota, and 1 member of Planctomycetes and Nitrospirae in G4.

The levels of most network indices were enhanced by T2 and T4 relative to T1 in the BGS, except MD and NC, in which all indices of the network except N, ACC, MD, and NC in T4 were lower than those in T2. However, the levels of most network indices were enhanced by G2 and G4 relative to G1 in the FDS, except MD and D, with most indices in G4 being higher than those in G2 except APL (average path length), ND (network diameter) and MD (Supplementary Figure S4, Supplementary Table S3).

There were 4, 24, and 19 hubs (>4 degrees) in CK, single species and mixed species of IAP in the BGS soil, respectively, mainly including Paecilomyces, Myrothecium, Monographella, and Arthrographis (Ascomycota), Aquicella (Proteobacteria) in CK, 7 members of Ascomycota, 6 members of Proteobacteria, 3 members of Basidiomycota, 2 members each of Actinobacteria and Bacteroidetes, 1 member each of Verrucomicrobia, Nitrospirae, Planctomycetes, and Firmicutes in single species and 6 members of Proteobacteria, 5 members of Bacteroidetes, 3 members of Zygomycota, and 2 members of Actinobacteria and Basidiomycotain in mixed species. However, there were 1, 20, and 19 hubs (>4 degrees) of CK, single species and mixed species of IAP in the FDS soil, respectively, mainly including 1 member of Chloroflexi (Roseiflexus) in CK; 6 members of Ascomycota, 4 members of Proteobacteria, 3 members of Actinobacteria, 1 member each of Firmicutes, Gemmatimonadetes, Zygomycota, Acidobacteria, Bacteroidetes, Basidiomycota, and Chloroflexi in the single species; 8 members of Ascomycota and 3 members each of Proteobacteria and Actinobacteria in the single species; and 1 member each of Acidobacteria, Firmicutes, Bacteroidetes, Basidiomycota, and Gemmatimonadetes in the mixed species.

### The Soil N and P Correlations With the Decomposition of SOC

**Figure 6:**
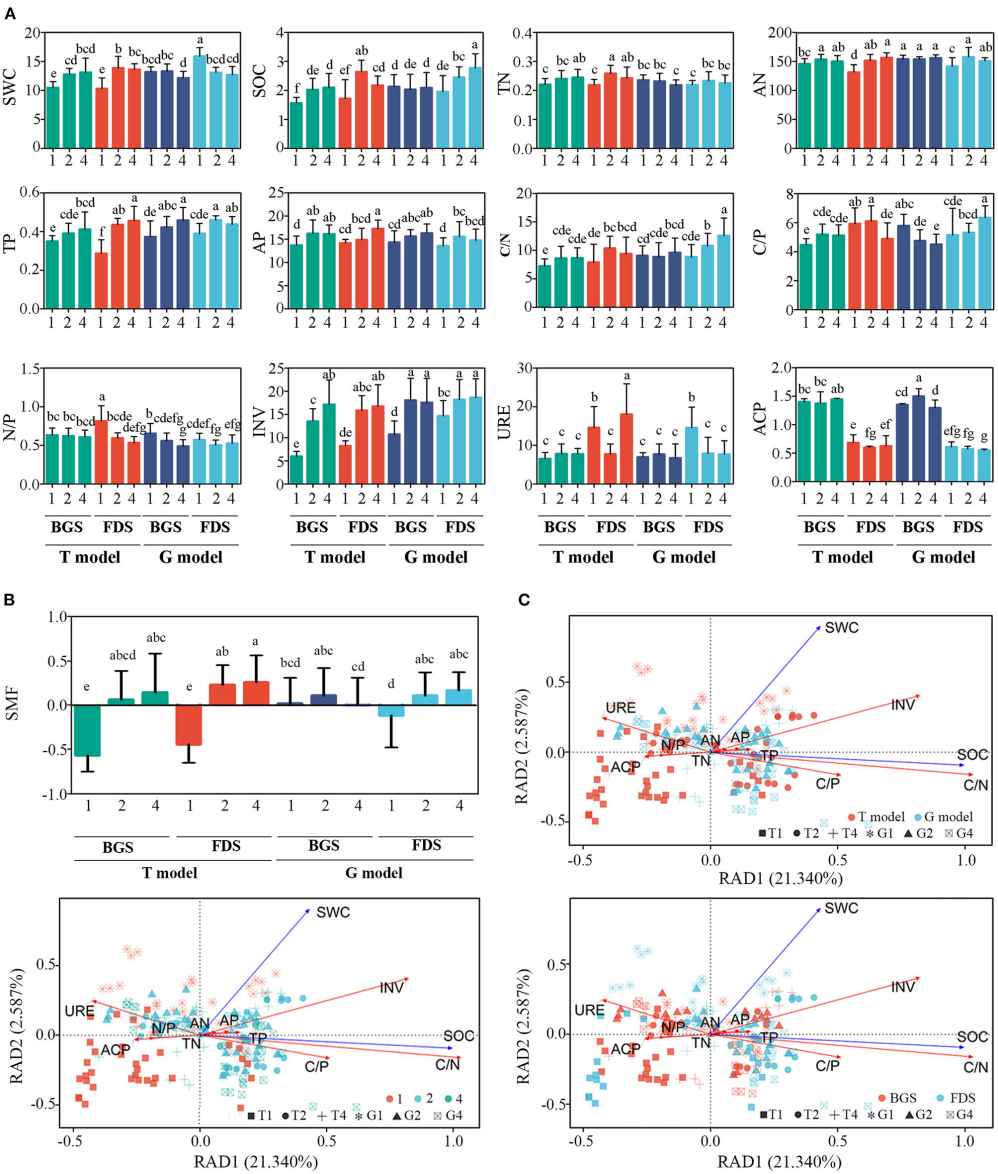
Variation in soil properties, SMF index and redundancy analysis (RDA) of soil nutrient variables between the intercropping patterns and between the development stages in the studied soils. **(A)** Variation in soil properties. **(B)** SMF, soil ecosystem multifunctionality. **(C)** RDA constrained by basic physico-chemical parameters. SWC, soil water content; SOC, total soil organic C content; TN, total nitrogen (g kg^−1^); TP, total phosphorus (g kg^−1^); AN, available nitrogen (mg kg^−1^); AP, available phosphorus (g kg^−1^); C/N, ratio between SOC and TN; C/P, ratio between SOC and TP; N/P, ratio between TN and TP; URE, urease activity (mg NH4+-N g^−1^ soil 24 h^−1^); INV, invertase activity (mg glucose g^−1^ soil h^−1^); ACP, acid phosphatase activity (g^−1^ h^−1^). 1, 2, and 4 indicate intercropping with 0, 1, and 3 species of aromatic plants, respectively, to facilitate regression analysis. BGS, branch growth stage; FDS, fruit development stage. T model, intercropping with aromatic plants in the clean tillage soil; G model, intercropping with aromatic plants in the natural grass soil. Different letters indicate significant differences at *P* < 0.05 based on Duncan's multiple range test.

As aromatic plant species richness increased, soil INV and URE activities and then SWC, SOC, TN, TP, AN, AP, and C/N significantly increased; however, the N/P ratio decreased, resulting in SMF being significantly enhanced in the T model. When both T2 and T4 improved most of the tested soil physical and chemical indices, T4 only increased INV activity in the BGS, and increased URE activity and AP content, while decreased SOC and C/P ratio in the FDS compared with T2. Similar to the T model, both G2 and G4 improved most of the soil indices, however, G4 decreased the SWC, ACP in the BGS and increased the SOC and C/N and C/P ratios in the FDS compared with G2 (Figure 6A). These results indicated that the activation of nutrients by the intraspecific interaction of aromatic plant species richness in the T model was significantly attenuated by the interspecific interaction between aromatic plant species richness and grass vegetation. With plant species richness, the SOC, TP, AP, and INV in the soil showed an increasing trend in the BGS, and the N/P ratio obviously decreased, resulting in an increasing trend in the SMF. Similarly, the SOC, TP, AP, AN, C/N ratio and INV activity showed significant increasing trends in the FDS, and the N/P ratio obviously decreased, resulting in an increasing trend of SMF with plant species richness (Figures 6A,B, Supplementary Figure S5).

Changes in soil abiotic factors driven by management and development stages resulted in a clear separation among the planting number levels in an ordination plot, which demonstrates that there were significant differences between management in dim1 and among planting numbers in dim2 (Figure 6), while there were no differences between development stages in dim1 and between the planting numbers in dim2. These changes were mainly driven by changes in SWC, SOC, the C/N and C/P ratios, and INV, URE and ACP activities.

## Discussion

### The Microbiome Properties and Nutrient Cycling Through Interspecific Interactions of Aromatic Plants in the T Model

Understorey vegetation plays an important role in maintaining soil nutrient cycling and microbial activity and promoting soil ecosystem diversity and stability (Ali and Yan, 2018; Oliveira et al., 2018; Ottaviani et al., 2019; Zhang et al., 2020b), which were likely caused by changing the diversity of aboveground vegetation and its litter composition (Cong et al., 2015; Ottaviani et al., 2019). In modern orchard production, the long-term application of pesticides and chemical fertilizers has led to degradation of soil fertility and quality, whereas selective introduction of functional plants can prevent this adverse effect and promote healthy growth and productivity of fruit trees (Hinsinger et al., 2011; Hunter et al., 2014; Sun et al., 2018; Zhang et al., 2020b). In our previous study, we found that single aromatic plant intercropping under a clean tillage pattern in a pear orchard improved the structure, diversity and N-related functional groups of the microbial community based on their root exudates and then increased N release (Zhang et al., 2021). However, the mechanism by which intercropping with various aromatic plant species under natural grass patterns regulates the microbial community to improve nutrient cycling remains limited. In the present study, we designed treatments with 0, 1, and 3 species of aromatic plants intercropped in two scenarios of clean tillage (T model, T1, T2, and T4) and natural grass (G model, G1, G2, and G4) in apple orchards, and we tested soil nutrient indices and analyzed both bacterial and fungal community characteristics in different development stages of the BGS and FDS to investigate how the introduction of aromatic plants, interspecific interactions between aromatic plants and intraspecific interactions between aromatic plants and natural grass mediate soil microbiota to improve nutrient cycling in orchards and differences between the patterns.

T4 enhanced the G^+^/G^−^ ratio in the BGS, while it enhanced the G^+^/G^−^ ratio and decreased the B/F ratio in the FDS (Figure 1). Similar experiments by Ainalidou et al. indicated that when tomato seedlings grow in soils enriched with spearmint and peppermint, the soil microbial community flourishes and microbial biomass increases (Ainalidou et al., 2021). Meanwhile, the structure of the total bacterial community at the phylum level in the BGS and those of the total fungal community at the order, family, and genus levels in the FDS were decreased by T4 (Figure 2A). This result suggested that the promotion of T2 to microbial biomass could be reduced by T4, suggesting that this may be caused by the root antibacterial components from interspecific interactions among aromatic plants alone or with fruit trees, the effect of different litter compositions, and more competition for soil resources for fruit development and aromatic intercropping plants than that for soil microbes (Bai et al., 2000). The produced antagonistic, non-additive effects on microbial biomass and structure of the total microbial community are mainly explained by the litter chemical composition and chemical diversity of mixed aromatic plants (Mao et al., 2017).

The alpha diversity index of the bacterial community in the FDS was enhanced by T4, and those of the fungal community in the BGS and FDS were promoted by T4 alone (Figure 2B, Supplementary Figure S2). The beta diversity of the two communities was distinguished (Figure 2C). This result suggested that aromatic plant intercropping in the orchard promoted the diversity of the microbial community to positively manipulate soil ecological functions and services, and intercropping with multispecies aromatic plant mixtures may also provide additional benefits by not only increasing microbial diversity but also enhancing the abundance of beneficial soil microbes compared with monocultures (Hamel et al., 2005; Mazzola and Manici, 2012; Bever et al., 2015) because of the positive relationship between plant biodiversity, availability, and diversity of root exudates, and soil microbial diversity (Garbeva et al., 2004; Maron et al., 2011; Fanin et al., 2014; Civitello et al., 2015).

With the increase from a single to three species of aromatic intercrops, the number of upregulated and downregulated core OTUs decreased and increased, respectively, which demonstrated the differences between the BGS and FDS (Figure 3). This result suggested that the soil core taxa of the two communities were possibly activated and inhibited by single and mixed intercropping aromatic plants, respectively, resulting in more downregulated OTUs by the intercrop mixture in different host developmental stages. Furthermore, T4 altered the relative abundance of a few bacterial taxa in the BGS and enhanced that of almost all dominant bacteria and their taxonomic members in the FDS (Figure 4). T4 altered the relative abundance of Ascomycota and Basidiomycota as well as their taxa. Tang et al. demonstrated that a mixture of peanut and sugarcane intercropping significantly increased the number of unique microbes and the relative abundance of bacteria and fungi in sugarcane soils compared with those in single planting fields (Tang et al., 2021). Beule et al. found that the composition of the bacterial communities in tree rows differed from those in crop rows of agroforestry systems and conventional monoculture croplands, in which the abundance of soil bacteria increased (Beule and Karlovsky, 2021). These effects occurred primarily because organic matter inputs of different cover crops stimulated the growth of dominant taxa and promoted fungal and bacterial alpha diversity, while the different qualities of plant residues stimulated the specific expression of soil microbial community structure and function (Wang et al., 2020). This variation in most microbial community groups was possible from the understorey herb biomass and herb species richness, as well as the tree biomass and cover of overstory trees, in which the understorey herb layer exerts strong controls on the soil microbial community in subtropical plantations (Yin et al., 2016). This result suggested that T4 reduced the core OTUs of two communities, such as Acidobacteria and Verrucomicrobia, when T2 promoted them, but it promoted the relative abundance of the dominant phylum, especially Proteobacteria and its members, in two communities, resulting in a balance of the core OTUs and relative abundance in the regulation of soil nutrient cycling.

Based on the properties of the co-occurrence network, including the bacterial and fungal communities, the levels of most network indices were reduced more by T4 than T2 (Figure 5, Supplementary Table S3), suggesting that the relationships of microbial communities were altered, and the complexity and stability of the co-occurrence network were reduced because of the interspecific interactions in the intercrop mixture. The keystone taxa often had higher relative abundances and fewer core OTUs, which may significantly impact network stability (Chen and Wen, 2021). The interspecific interactions of peanut and sorghum, and wheat and faba bean were also helpful for maintaining the stability and ecological functions of microbial communities by restructuring the otherwise stable core microbiome. This was because the number of negative interdomain correlations between fungi and bacteria decreased in mixed intercropping compared with monocultures due to beneficial effects (Sandra et al., 2017; Shi et al., 2021). However, various plant species-dependent aromatic plants demonstrated that intraspecies and interspecific competition between plants had varied effects on the plant species and, therefore, on their associated microbial communities (Sandra et al., 2017).

Moreover, we also found that as aromatic plant species richness increased, soil enzyme activities and C, N, and P nutrients significantly increased, resulting in significantly enhanced SMF (Figures 6A,B, Supplementary Figure S5). The soil nutrient framework could be beneficial to tree growth and fruit development. Many studies have demonstrated that intercropping in orchards promotes soil SOM content and SOM degradation-related enzymes and enhances N and P availability by increasing N- and P-related enzyme activities, resulting in an increase in fruit production (Cui et al., 2015; Gao et al., 2019; Liu et al., 2019; Zheng et al., 2019). These effects could not be decreased, although the mixed intercropping with aromatic plants could lead to a decrease in microbial biomass, core OTUs, abundance, network complexity and stability compared with monoplanting due to their litter chemical diversity through interspecific interactions of aromatic plants, which often contain various unique organic components, such as ocimene, linalool, patuletin, and menthol (Wahbi et al., 2016; Mao et al., 2017; Misra et al., 2019; Tang et al., 2021).

### The Microbiome Properties and Nutrient Cycling According to Intraspecific Interactions of Aromatic Plants in the G Model

Compared with T1, G1 promoted the most PLFA indices but did not change the B/F and G^+^/G^−^ ratios or the percentage of taxa in the total bacterial community in the BGS and FDS, while a low percentage of the fungal community was observed in the FDS due to the vegetation diversity of nature grass (Figure 1). G1 simultaneously enhanced alpha and beta diversities of bacterial community in FDS, and the relative abundance of bacteria community at order and family levels in BGS but reduced that of the fungal community in the FDS (Figure 2), possibly resulting from the effect of mixed litter of natural grass vegetation and fruit tree root special exudates in the FDS (Cong et al., 2015; Mao et al., 2017). This effect of natural grass vegetation in the G model was similar to that of monoplanting aromatic plants in the T model. Additionally, the topological parameters of the co-occurrence networks in G1 soil were greater than those in T1 (Figure 5, Supplementary Table S3), indicating more complexity and stability of the microbial community in G1 soil. These characteristics were linked with more C and N nutrients as well as the SMF index in the BGS and FDS (Figure 6). These results are consistent with those of many previous studies (Bardgett and Wardle, 2003; Bever et al., 2015), suggesting that the diversity of understorey vegetation was beneficial to the microbial biomass, taxonomic structure and abundance, diversity index and network parameter of the microbial community, promoting the stability and nutrient enrichment of the soil ecosystem.

G4 enhanced the biomass of total, bacteria and G^+^ in the BGS and reduced all PLFAs in the FDS to a greater extent than G2 (Figure 1). This result suggested that differences between single and mixed intercropping to alter the composition of PLFAs were mitigated not only in the BGS when PLFAs were higher in the G model than in the T model but also in the FDS when PLFAs were lower in the G model than in the T model due to the interaction of intercropped aromatic plants with the grass nature vegetation and host development stage (de Vries et al., 2006; Thiet et al., 2006). Batten et al. found that after introducing Centaurea solstitialis and Aegilops triuncialis to native plants, the number of sulfate-reducing bacteria, sulfur-oxidizing bacteria and arbuscular mycorrhizal fungi increased (Batten et al., 2006). G4 increased the percentage of all bacterial taxa (except phyla) in the BGS, which was significantly different from the result of the T model but was associated with PLFA changes (Figure 2A). This result suggested that the impact on the structure of the two communities was more obvious in mixed intercropping than in single intercropping, with a contrary tendency, and did not always reduce the community structure ratio as occurred in the T model, which possibly regulated the structure of the total microbial community by altering the taxonomic structure of the bacterial and fungal communities (Saggar et al., 1999; Niu et al., 2007).

The alpha diversity of bacterial community in the G model was higher than that in the T model in the FDS. G4 increased Sob and Chao in the bacterial community in the BGS and maintained alpha diversity in the FDS and the Shannon index in the fungal community. These changes in the G model were almost the opposite of those in the T model (Figure 2B, Supplementary Figure S2). The beta diversity of the bacterial community was distinguished and was more remarkable than that of the fungal community, while the differences between the G2 and G4 regarding beta diversity were more distinct in the fungal community than in the bacterial community. These changes in the G model were similar in the bacterial community and contrary in the fungal community to those in the T model (Figure 2C). This result suggested that the bacterial community was more sensitive to aromatic plant intercropping, while the fungal community was more sensitive to monocultures and mixtures of aromatic plants, possibly due to the preference effects of vegetation diversity on the bacterial community and the effects of interspecific competition on the fungal community. Lekberg et al. found that the introduction of Centaurea stoebe and Euphorbia esula enhanced AMF richness and diversity (Lekberg et al., 2013). These characteristics are associated with soil nutrient cycling (Xiao et al., 2014).

Compared with the T model, the G model significantly increased the numbers of upregulated and downregulated OTUs in the two communities. With the increase in aromatic intercrop species, the upregulated number of core OTUs increased in the BGS and decreased in the FDS, while the downregulated number of core OTUs showed the opposite trend (Figure 3). Correspondingly, G4 promoted the relative abundance of more dominant microbial taxa in the BGS (Figure 4). These results basically corresponded to the change in PLFA biomass, structure proportion and diversity of the two communities. Chen et al. showed that introducing *Mikania micrantha* and *Bidens pilosa* altered the relative abundance of α-, β-, and γ-Proteobacteria and their related dominant bacteria (Batten et al., 2006; Chen et al., 2011). Xiao et al. found that soil fungi rather than bacteria were modified by introducing plants and that which benefited the introduction of plant growth (Xiao et al., 2014). We speculate that invasive grass dominance over these natives may be partially due to effects on the rhizosphere community, with changes in specific bacterial families potentially benefiting the introduced plants at the expense of natives (Laforgia et al., 2021).

The network indices and hubs in the G model were significantly higher than those in the T model, in which the levels of most network indices and hubs were enhanced by G4 (Figure 5, Supplementary Table S3). This result suggested that G4 possibly neutralized the relationships of keystone taxa of microbes and improved the network complexity and stability of the microbial community (Chen and Wen, 2021). As indicated by the fungal Bray-Curtis and weighted UniFrac distances, the fungal community homogenized from G2 to G4. This was also because G4 had a higher proportion of saprophytic fungi, which benefits higher organic matter decomposition (Zhang et al., 2020). Similar to the T model, G4 improved most of the soil nutrient indices (Figure 6A, Supplementary Figure S5). Earlier research has shown that the abundance of bacterial N-cycling functional guilds varied under teak and mixed species plantations and that interplanting teak with flueggea may potentially alleviate N losses associated with nitrification and denitrification and favor N retention. Mixed plantations could also allow an increase in soil C and N stocks without losing the source of income that teak trees provide for local communities (Saggar et al., 1999; Reverchon et al., 2015).

### The Effects of the Developmental Stages of Hosts and Intercrops on Microbiome Properties and Nutrient Cycling

We found that the effects of developmental stages of the fruit trees on the PLFAs were generally higher than those in the models, and differences between the BGS and FDS in the G model were greater than those in the T model (Figure 1), suggesting that the developmental stage and intercropping patterns had significant coregulatory effects on the PLFAs. The percentage of taxa in the total bacterial community in the FDS was lower than that in the BGS in the G model, in contrast to that in the T model. The percentage of the fungal community in T4 in the FDS was lower than that in the BGS in the T model, while the percentage of G4 in the FDS was not different from that in the BGS in the G model (Figure 2A). The alpha diversity of the bacterial community in the FDS was higher than that in the BGS in the two models, except for the Shannon index in the G model. The alpha diversity of the fungal community was higher in the BGS than in the FDS in the T model, as was G4 in the G model (Figure 2B). The beta diversity of the bacterial community showed small differences between the FDS and BGS, while the beta diversity of the fungal community showed large differences between the two stages and among G1, G2, and G4 (Figure 2C). Differences in diversity in the microbial community have been observed in many studies among rhizosphere samples across different crop development stages. Plant growth promoting bacteria (PGPR), such as Bacillus, were enriched during the middle stages of crop development, but there was a decline in PGP organisms in the mature crop stage (Hinsu et al., 2021).

The numbers of significantly different OTUs of the bacterial and fungal communities in the FDS were higher than those in the BGS in the two models, except fungal community in the T model, which showed significant differences between the models and between the single and mixed intercropping systems (Figure 3). Correspondingly, the changes in the relative abundance of dominant taxa were higher in the FDS than in the BGS in the T model, while the opposite was true in the G model (Figure 4). Most network indices of CK (T1 and G1) and mixed intercropping (T4 and G4) were higher in the BGS than in the FDS, but those of single intercropping (T2 and G2) were the opposite. The hub numbers of the microbial communities were not different between the BGS and FDS, although those in the BGS were higher than those in the FDS (Supplementary Figure S4, Supplementary Table S4). This result suggested that developmental stages affect the complexity and stability of the microbial community. Most of the C and N nutrient contents were higher in the FDS than in the BGS, especially in the G model (Figure 6A, Supplementary Figure S4). These may be beneficial to fruit development and quality improvement in the latter part of the fruit tree's annual cycle.

Based on the effects of host developmental stage on the soil microbial community and nutrient cycling, we speculated that the composition and quantity of root exudates from hosts and intercrops play an important role in the regulation of the microbial community and nutrients at different developmental stages. Gschwendtner et al. found that the significant differences among potato cultivars and various developmental stages in rhizosphere microbial communities were associated with the composition and quantity of root exudates (Gschwendtner et al., 2011). Chaparro et al. found that the composition and concentration of the root exudates in Arabidopsis thaliana change significantly with growth and developmental stages, in which the genes related to root exudate metabolism correspondingly change. The functional gene expression profiles of the rhizosphere microbiome were correlated with the patterns of root exudate secretion during developmental processes (Chaparro et al., 2013). This is because plants can constantly adjust the composition and quantity of their root exudates according to the changes in the surrounding environment to respond positively.

Overall, the introduction of functional plants, such as aromatic plants, can increase primary production, change the chemical traits of crop residues (e.g., N and P concentrations) (Fageria et al., 2005; Maltais et al., 2014), and alter root traits (e.g., nitrogen-fixing bacteria and mycorrhizal fungi) (Möller et al., 2008; Turrini et al., 2016). These differences in functional traits can exert a considerable influence on agroecosystem functions (e.g., C sequestration and nutrient regulation) and in turn enhance the quality, quantity, and chemical diversity of C inputs while reducing leaching and erosion (Tiemann et al., 2015; Grabau and Chen, 2016; Li et al., 2018). These factors may modulate the soil microbial community diversity and structure and further affect the formation and storage of SOC (Taheri et al., 2016; Bakhshandeh et al., 2017; Pausch and Kuzyakov, 2018).

## Conclusions

We found that G model promoted diversity, relative abundance of dominant taxon, and indices of co-occurrence network, then promoted soil C, N, and P content relative to T model. In G model, alpha diversity of microbial community was lower, while the relative abundance of Acidimicroiia, Acidimicrobiales, and Spartobacteria and the most of network indices were higher in G2 and G4 than those in G1. Although the range of these increases were lower in G4 than those in G2, the nutrient content of G4 was higher than that in G2. Our results suggested that intercropping with mixture aromatic plants regulated microbial community characteristic and then improved soil nutrient cycling, benefit further for apple tree growth and fruit quality during the fruit development period. There is mechanism in the chemical diversity of litter from mixed species of aromatic plants and natural grass to soil microbiome and nutrient cycling. It will be note that the pesticides and chemical fertilizers have been widely used for a long time, resulting in soil degradation, harmful pollution, successive cropping obstacle and output losses on orchard (Kumar et al., 2018). Most orchards in north China adopt natural grass management model year after year, which may limit land use efficiency and farmer income in some areas. Importantly the effects of this model on control of pest, regulation of soil microbial community and improvement of soil nutrient cycling were limited (Song et al., 2013). Therefore, it is crucial to explore a suitable management model *via* selecting advantages species of aromatic plants and even other plant species to construct compound ecological system in orchard.

## Data Availability Statement

The data presented in the study are deposited in the NCBI SRA database, accession number PRJNA813412.

## Author Contributions

YS, LC, SZ, YM, JZhao, and LY: data curation. YY: funding acquisition. YS, LC, SZ, YM, YZ, ZL, JZhao, LY, JZhan, and XQ: experiments. YY, YS, and XQ: writing-original draft. YY and YS: writing-review and editing. All authors contributed to the article and approved the submitted version.

## Funding

Financial support was provided by National Key Project of Research and Development Plan (2016YFD0201116), Scientific Research Program of Beijing Municipal Commission of Education (KM202110020007).

## Conflict of Interest

The authors declare that the research was conducted in the absence of any commercial or financial relationships that could be construed as a potential conflict of interest.

## Publisher's Note

All claims expressed in this article are solely those of the authors and do not necessarily represent those of their affiliated organizations, or those of the publisher, the editors and the reviewers. Any product that may be evaluated in this article, or claim that may be made by its manufacturer, is not guaranteed or endorsed by the publisher.

## Supplementary Material

Supplementary Figure S1 Schematics of the intercropping of different aromatic plants in the field experiment. **(A)** Schematic of each plot; **(B)** Schematic of two models with three treatments; **(C)** Schematic of five sampling points in each plot. The different treatments were separated from adjacent plots by a 10 m isolation belt. 1, 2, and 4 indicate intercropping with 0, 1, and 3 species of aromatic plants, respectively, to facilitate regression analysis. T model, intercropping with aromatic plants in the clean tillage soil; G model, intercropping with aromatic plants in the natural grass soil. BGS, branch growing stage; FDS, fruit development stage.

Supplementary Figure S2 Linear least-squares regression relationships between the plant species richness and alpha diversity of the microbial community in the studied soil. **(A)** Alpha diversity of the bacterial and fungal communities between intercropping patterns. **(B)** Alpha diversity of bacterial and fungal communities between development stages. The adjusted R2 value was used to determine whether the models were fitted with the species richness of intercropping with aromatic plants. The lines denote the least-squares linear regressions across species richness, with their 95% confidence intervals (gray-shaded areas). “y,” regression equations of the fitting line; ^*^*P* < 0.05; ^**^*P* < 0.01; ^***^*P* < 0.001. 1, 2, and 4 indicate intercropping with 0, 1, and 3 species of aromatic plants, respectively, to facilitate regression analysis. T model, intercropping with aromatic plants in the clean tillage soil; G model, intercropping with aromatic plants in the natural grass soil.

Supplementary Figure S3 Linear least-squares regression relationships between the plant species richness and similarity of the both bacterial and fungal community in the studied soil between intercropping patterns and between development stages. The adjusted R2 value was used to determine whether the models were fitted with the species richness of intercropping with aromatic plants. The lines denote the least-squares linear regressions across species richness, with their 95% confidence intervals (gray-shaded areas). “y,” regression equations of the fitting line; ^*^*P* < 0.05; ^**^*P* < 0.01; ^***^*P* < 0.001. 1, 2, and 4 indicate intercropping with 0, 1, and 3 species of aromatic plants, respectively, to facilitate regression analysis. T model, intercropping with aromatic plants in the clean tillage soil; G model, intercropping with aromatic plants in the natural grass soil; BGS, branch growing stage; FDS, fruit development stage.

Supplementary Figure S4 Co-occurrence network of the microbial community between the development stages. 1, 2, and 4 indicate intercropping with 0, 1, and 3 species of aromatic plants, respectively, to facilitate regression analysis. BGS, branch growing stage; FDS, fruit development stage.

Supplementary Figure S5 Variation in soil properties between the intercropping patterns **(A)** and between the development stages **(B)** in the studied soils. SWC, soil water content; SOC, total soil organic C content; TN, total nitrogen (g kg^−1^); TP, total phosphorus (g kg^−1^); AN, available nitrogen (mg kg^−1^); AP, available phosphorus (g kg^−1^); C/N, ratio between SOC and TN; C/P, ratio between SOC and TP; N/P, ratio between TN and TP; URE, urease activity (mg NH4+-N g^−1^ soil 24 h^−1^); INV, invertase activity (mg glucose g^−1^ soil h^−1^); ACP, acid phosphatase activity (g^−1^ h^−1^). 1, 2, and 4 indicate intercropping with 0, 1, and 3 species of aromatic plants, respectively, to facilitate the regression analysis. The adjusted R2 was used to determine whether the models were fitted with the species richness of intercropping with aromatic plants. The lines denote the least-squares linear regressions across species richness, with their 95% confidence intervals (gray-shaded areas). “y,” regression equations of the fitting line; ^*^*P* < 0.05; ^**^*P* < 0.01; ^***^*P* < 0.001. 1, 2, and 4 indicate intercropping with 0, 1, and 3 species of aromatic plants, respectively, to facilitate regression analysis. T model, intercropping with aromatic plants in the clean tillage soil; G model, intercropping with aromatic plants in the natural grass soil.

Supplementary Table S1 The total bacterial community composition at the phylum levels in soil with different treatment.

Supplementary Table S2 The total fungal community composition at the phylum levels in soil with different treatment.

Supplementary Table S3 Network properties of soil microbial communities in different pattern.

Supplementary Table S4 Network properties of soil microbial communities according to development stage.
