# Supplementary figures and images for: Plant Interaction Patterns Shape the Soil Microbial Community and Nutrient Cycling in Different Intercropping Scenarios of Aromatic Plant Species
Source: Front Microbiol. 2022 May 27;13:888789. doi: 10.3389/fmicb.2022.888789 (PMC9197114; doi:10.3389/fmicb.2022.888789)

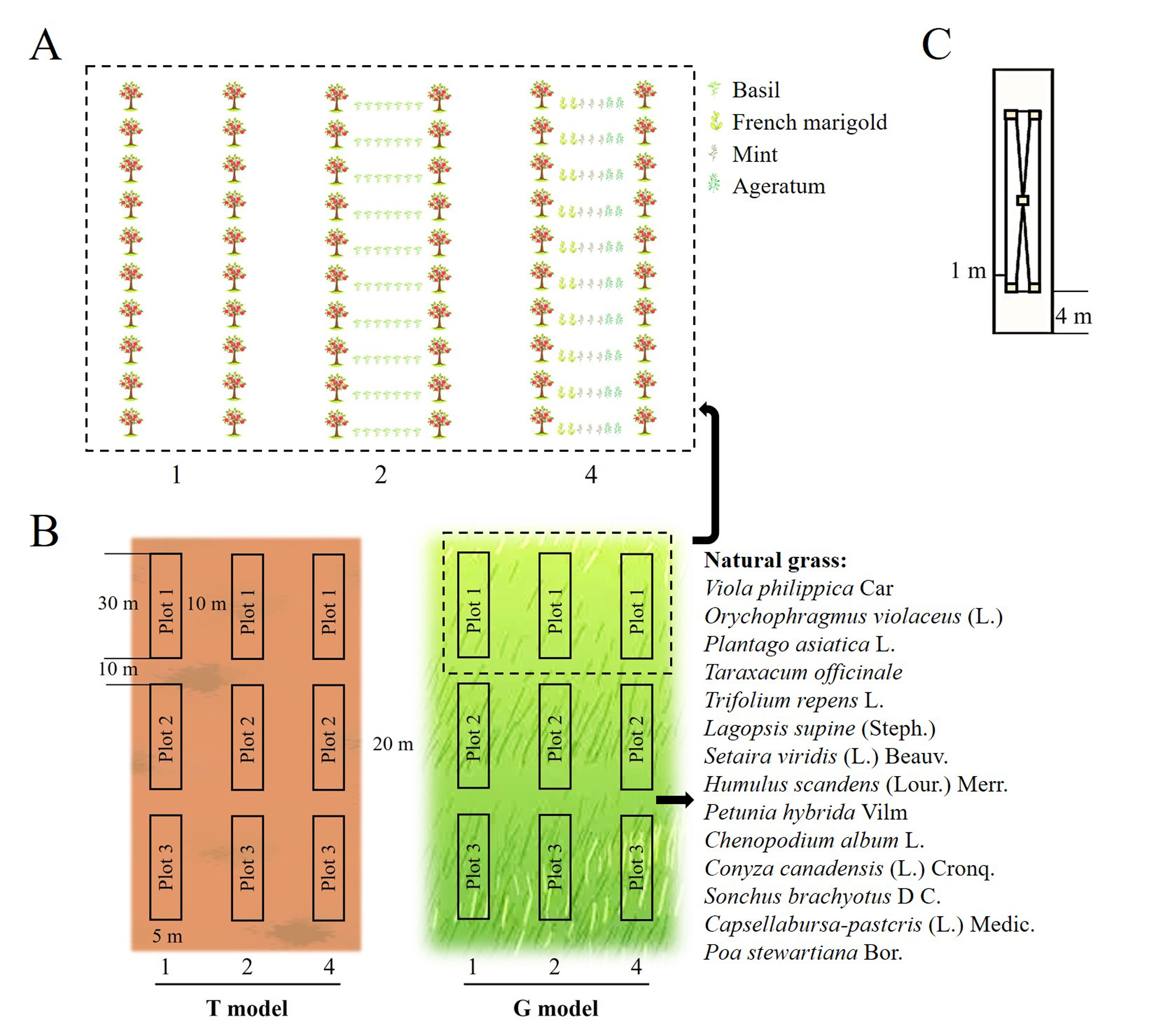

Supplement: Supplementary Figure S1 — Schematics of the intercropping of different aromatic plants in the field experiment. (A) Schematic of each plot; (B) Schematic of two models with three treatments; (C) Schematic of five sampling points in each plot. The different treatments were separated from adjacent plots by a 10 m isolation belt. 1, 2, and 4 indicate intercropping with 0, 1, and 3 species of aromatic plants, respectively, to facilitate regression analysis. T model, intercropping with aromatic plants in the clean tillage soil; G model, intercropping with aromatic plants in the natural grass soil. BGS, branch growing stage; FDS, fruit development stage. [file Image_1.TIF]

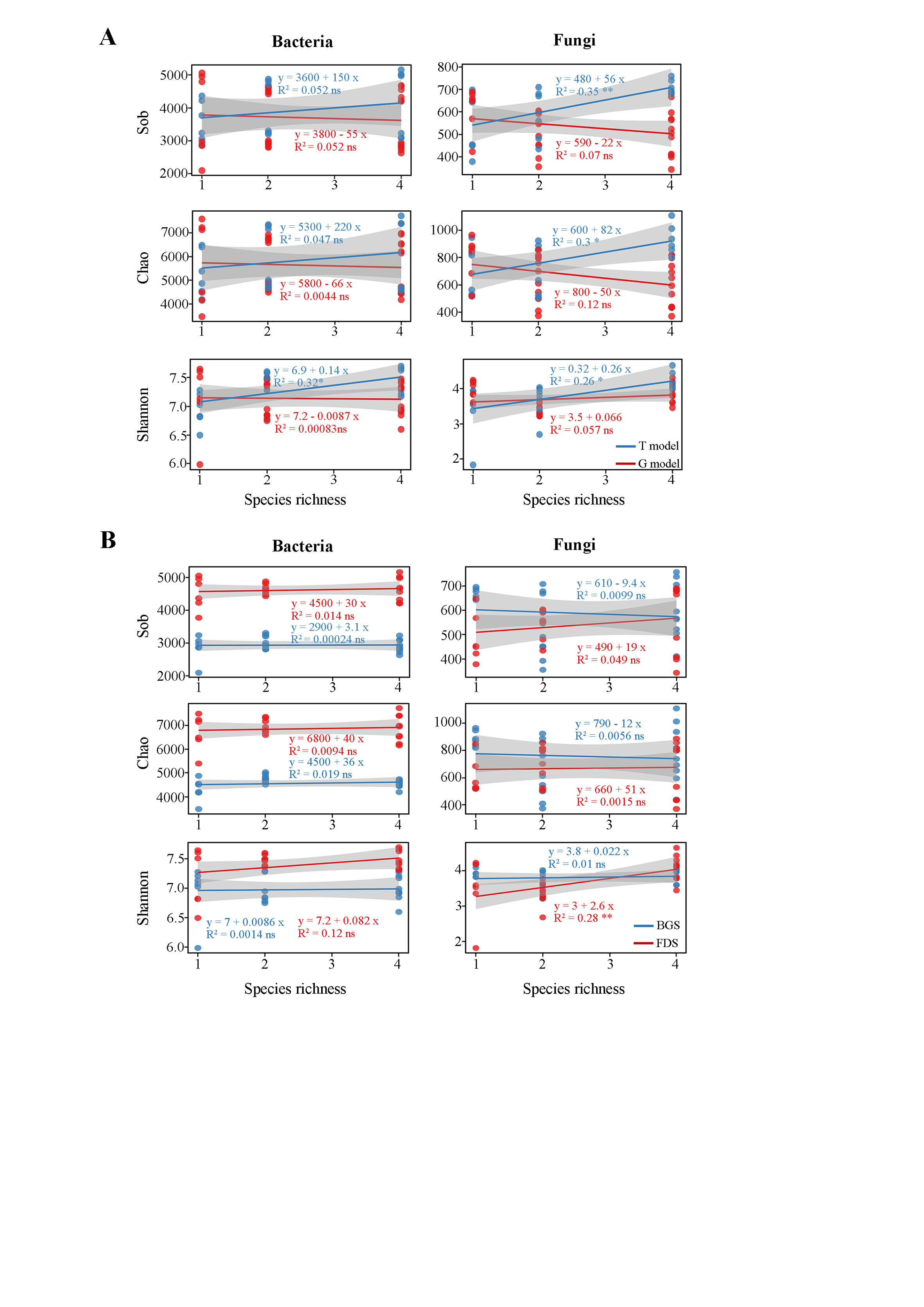

Supplement: Supplementary Figure S2 — Linear least-squares regression relationships between the plant species richness and alpha diversity of the microbial community in the studied soil. (A) Alpha diversity of the bacterial and fungal communities between intercropping patterns. (B) Alpha diversity of bacterial and fungal communities between development stages. The adjusted R2 value was used to determine whether the models were fitted with the species richness of intercropping with aromatic plants. The lines denote the least-squares linear regressions across species richness, with their 95% confidence intervals (gray-shaded areas). “y,” regression equations of the fitting line; *P < 0.05; **P < 0.01; ***P < 0.001. 1, 2, and 4 indicate intercropping with 0, 1, and 3 species of aromatic plants, respectively, to facilitate regression analysis. T model, intercropping with aromatic plants in the clean tillage soil; G model, intercropping with aromatic plants in the natural grass soil. [file Image_2.TIF]

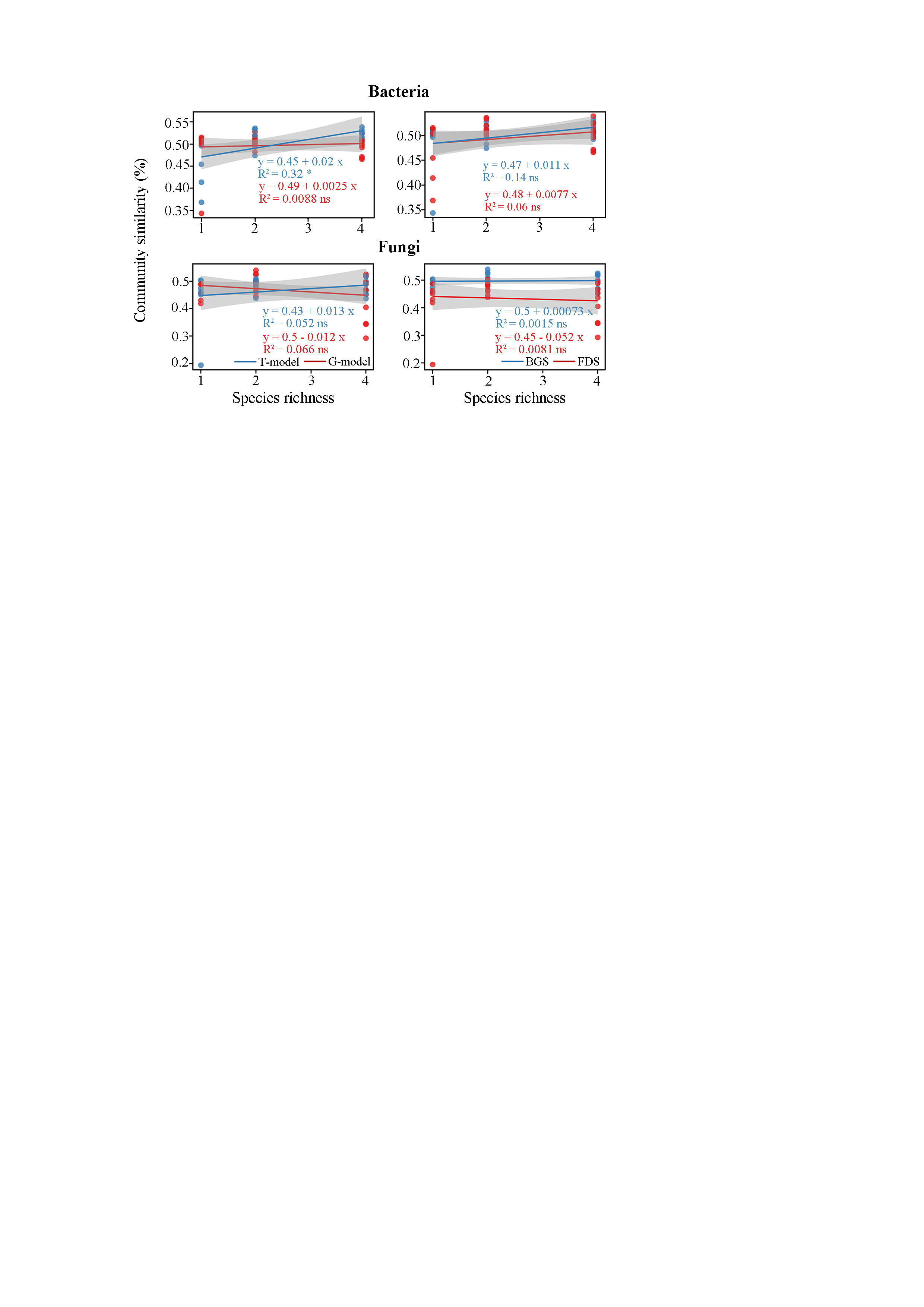

Supplement: Supplementary Figure S3 — Linear least-squares regression relationships between the plant species richness and similarity of the both bacterial and fungal community in the studied soil between intercropping patterns and between development stages. The adjusted R2 value was used to determine whether the models were fitted with the species richness of intercropping with aromatic plants. The lines denote the least-squares linear regressions across species richness, with their 95% confidence intervals (gray-shaded areas). “y,” regression equations of the fitting line; *P < 0.05; **P < 0.01; ***P < 0.001. 1, 2, and 4 indicate intercropping with 0, 1, and 3 species of aromatic plants, respectively, to facilitate regression analysis. T model, intercropping with aromatic plants in the clean tillage soil; G model, intercropping with aromatic plants in the natural grass soil; BGS, branch growing stage; FDS, fruit development stage. [file Image_3.TIF]

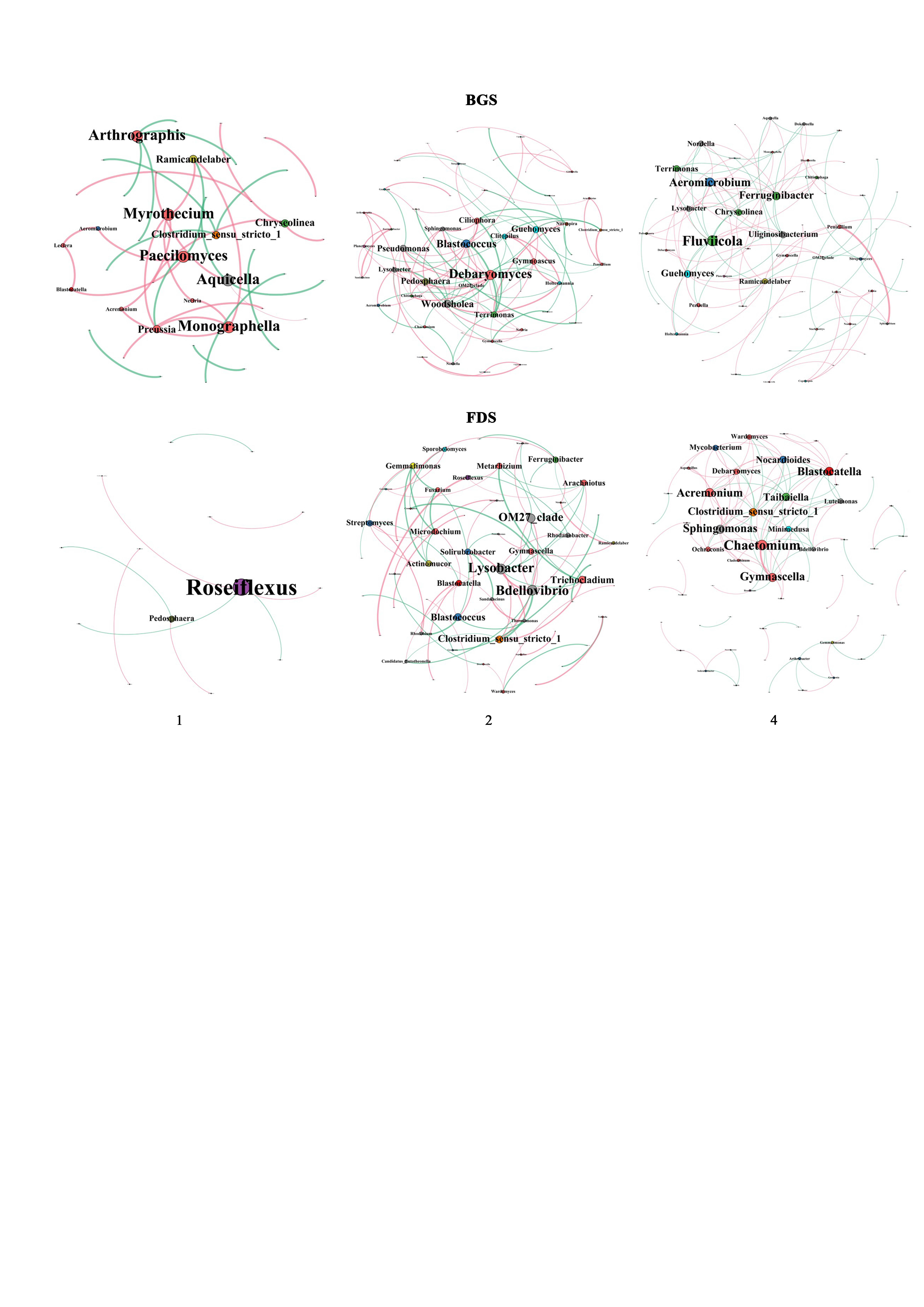

Supplement: Supplementary Figure S4 — Co-occurrence network of the microbial community between the development stages. 1, 2, and 4 indicate intercropping with 0, 1, and 3 species of aromatic plants, respectively, to facilitate regression analysis. BGS, branch growing stage; FDS, fruit development stage. [file Image_4.TIF]

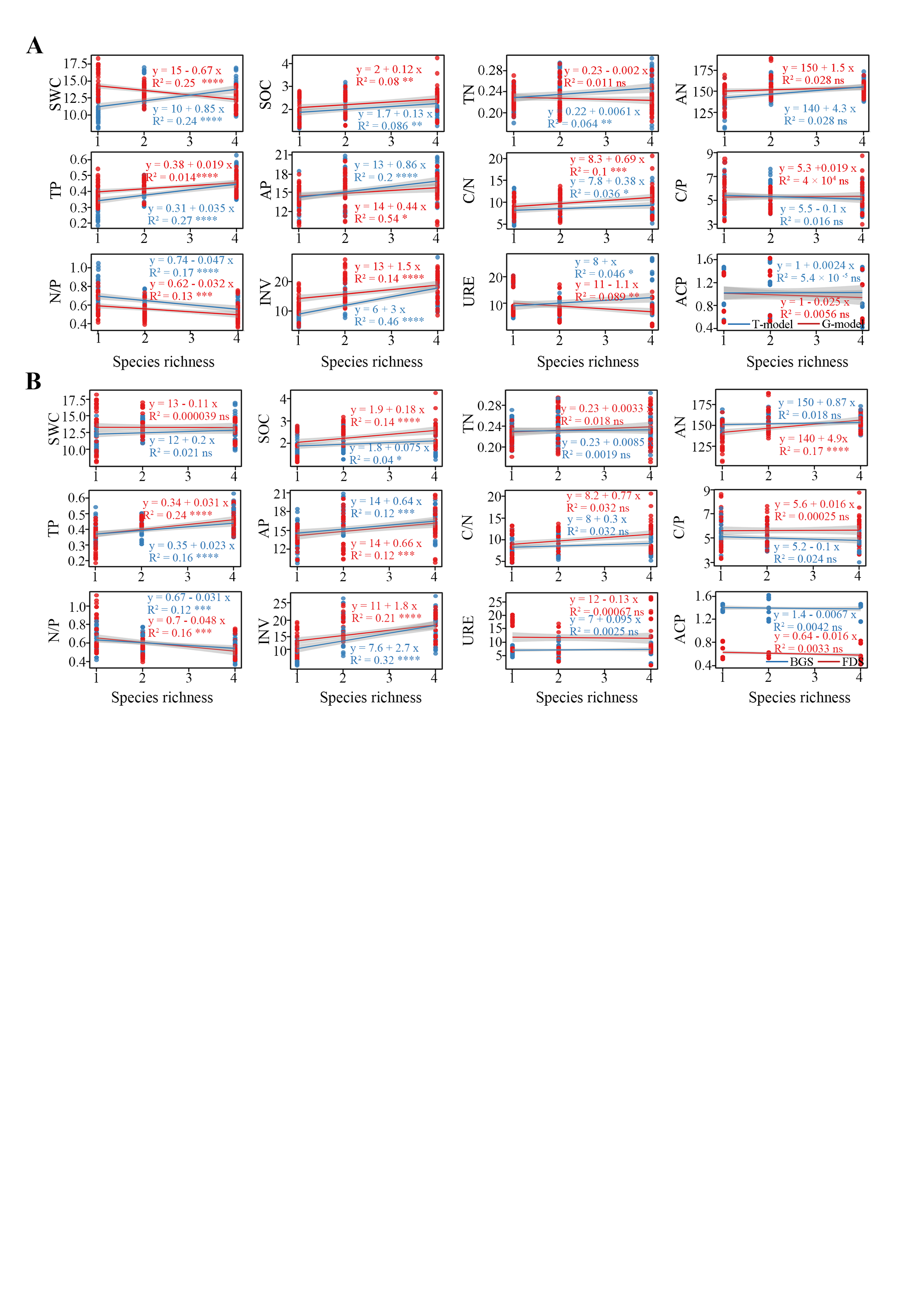

Supplement: Supplementary Figure S5 — Variation in soil properties between the intercropping patterns (A) and between the development stages (B) in the studied soils. SWC, soil water content; SOC, total soil organic C content; TN, total nitrogen (g kg−1); TP, total phosphorus (g kg−1); AN, available nitrogen (mg kg−1); AP, available phosphorus (g kg−1); C/N, ratio between SOC and TN; C/P, ratio between SOC and TP; N/P, ratio between TN and TP; URE, urease activity (mg NH4+-N g−1 soil 24 h−1); INV, invertase activity (mg glucose g−1 soil h−1); ACP, acid phosphatase activity (g−1 h−1). 1, 2, and 4 indicate intercropping with 0, 1, and 3 species of aromatic plants, respectively, to facilitate the regression analysis. The adjusted R2 was used to determine whether the models were fitted with the species richness of intercropping with aromatic plants. The lines denote the least-squares linear regressions across species richness, with their 95% confidence intervals (gray-shaded areas). “y,” regression equations of the fitting line; *P < 0.05; **P < 0.01; ***P < 0.001. 1, 2, and 4 indicate intercropping with 0, 1, and 3 species of aromatic plants, respectively, to facilitate regression analysis. T model, intercropping with aromatic plants in the clean tillage soil; G model, intercropping with aromatic plants in the natural grass soil. [file Image_5.TIF]
