# Supplementary material for: Plant Interaction Patterns Shape the Soil Microbial Community and Nutrient Cycling in Different Intercropping Scenarios of Aromatic Plant Species
Source: Front Microbiol. 2022 May 27;13:888789. doi: 10.3389/fmicb.2022.888789 (PMC9197114; doi:10.3389/fmicb.2022.888789)
Supplement: Supplementary Table S1 — The total bacterial community composition at the phylum levels in soil with different treatment. [file Data_Sheet_1.PDF]

Table S1 | The total bacterial community composition at the phylum levels in soil with different treatment.

Phylum	T model						G model					
	BGS			FDS			BGS			FDS		
	1	2	4	1	2	4	1	2	4	1	2	4
Proteobacteria	26.03 ± 1.00 e	26.06 ± 0.31 e	25.99 ± 0.69 e	44.19 ± 2.17 a	26.60 ± 0.66 cde	29.36 ± 1.89 b	25.98 ± 1.96 e	26.28 ± 0.33 de	27.94 ±1.49 bcd	28.32 ±0.53 e	25.41 ± 0.40 e	25.24 ±0.86 e
Acidobacteria	12.14 ± 0.75 c	10.76 ± 0.61 cd	11.75 ± 0.16 c	11.92 ± 2.94 c	18.86 ± 0.94 a	17.16 ±0.28 b	11.90 ± 1.42 c	9.47 ± 0.37 d	10.88 ± 0.95 cd	19.51 ± 2.54 a	10.43 ± 0.55 cd	10.87 ± 0.32 cd
Bacteroidetes	10.74 ± 0.70 abc	10.99 ± 0.47 ab	11.42 ± 0.38 ab	9.15 ± 2.65 def	8.40 ± 0.49 f	8.31 ± 1.10 f	12.02 ±0.70 a	10.16 ± 0.37 bcde	10.47 ± 0.42 bcd	8.95 ± 0.52 ef	9.63 ± 0.27 cdef	9.48 ± 0.47 cdef
Actinobacteria	9.05 ± 0.05 abcd	9.00 ± 0.18 abcd	9.66 ± 0.50 ab	3.06 ± 0.53 f	9.57 ± 1.07 abc	8.91 ± 1.69 abcde	8.32 ± 1.04 bcde	9.98 ± 0.56 a	9.43 ± 0.55 abc	7.41 ± 2.77 e	7.46 ± 0.28 de	7.99 ± 0.34 cde
Planctomycetes	8.68 ± 0.27 ab	9.10 ± 0.17 a	8.85 ± 0.39 ab	3.86 ± 0.81 g	6.60 ± 0.48 f	8.51 ± 1.21 abc	8.34 ± 1.86 abcd	8.39 ± 0.51 abcd	7.84 ± 0.56 bcde	6.71 ± 0.95 ef	7.24 ± 0.23 def	7.38 ± 0.16 cdef
Gemmatimonadetes	4.99 ± 0.73 abc	4.39 ± 0.21 cde	4.54 ± 0.17 bcde	3.59 ± 0.43 f	4.38 ± 0.28 cde	4.15 ± 0.34 ef	5.06 ±0.59 abc	5.51 ± 0.17 a	4.96 ± 0.47 abcd	4.26 ± 0.17 def	5.03 ± 0.11 abc	5.21 ± 0.73 ab
Verrucomicrobia	5.51 ± 0.28 ab	5.07 ± 0.15 bc	5.10 ± 0.49 bc	4.01 ± 0.25 d	5.99 ± 0.08 a	5.15 ± 0.14 bc	5.22 ± 0.39 ab	3.67 ± 0.42 d	4.38 ± 0.62 cd	5.53 ± 0.39 ab	4.41 ± 0.85 cd	3.71 ± 0.26 d
Chloroflexi	3.13 ± 0.06 b	2.83 ± 0.22 bc	2.40 ± 0.30 cd	1.66 ± 0.38 e	2.81 ± 0.32 bc	2.91 ± 0.35 b	3.11 ± 0.17 b	2.24 ± 0.07 d	2.35 ± 0.25 d	2.14 ± 0.81 d	3.88 ± 0.11 a	3.80 ± 0.15 a
Firmicutes	1.19 ± 0.28 bc	1.36 ± 0.24 b	1.11 ± 0.04 bcd	0.47 ± 0.37 fg	0.55 ± 0.08 efg	0.45 ± 0.10 fg	0.97 ± 0.30 bcd	0.93 ± 0.12 cde	0.76 ± 0.07 def	0.25 ± 0.03 g	2.46 ± 0.38 a	2.51 ± 0.32 a
Nitrospirae	0.55 ± 0.07 cd	0.39 ± 0.05 d	0.96 ± 0.39 ab	0.62 ± 0.30 cd	0.75 ± 0.07 bc	0.59 ± 0.05 cd	0.40 ± 0.22 d	1.24 ± 0.28 a	1.19 ± 0.29 a	0.83 ± 0.03 bc	0.31 ± 0.02 d	0.31 ± 0.03 d

1, 2 and 4 indicate intercropping with 0, 1 and 3 species of aromatic plants, respectively, to facilitate regression analysis. T model, intercropping with aromatic plants in the clean tillage soil; G model, intercropping with aromatic plants in the natural grass soil. BGS, branch growth stage; FDS, fruit development stage.
