# Supplementary material for: Plant Interaction Patterns Shape the Soil Microbial Community and Nutrient Cycling in Different Intercropping Scenarios of Aromatic Plant Species
Source: Front Microbiol. 2022 May 27;13:888789. doi: 10.3389/fmicb.2022.888789 (PMC9197114; doi:10.3389/fmicb.2022.888789)
Supplement: Supplementary Table S2 — The total fungal community composition at the phylum levels in soil with different treatment. [file Data_Sheet_2.PDF]

Table S2 | The total fungal community composition at the phylum levels in soil with different treatment.

Phylum	T model						G model					
	BGS			FDS			BGS			FDS		
	1	2	4	1	2	4	1	2	4	1	2	4
Ascomycota	37.79 ± 0.84 abc	41.22 ± 2.22 a	44.37 ± 7.75 a	28.96 ± 15.66 bcd	25.12 ± 8.53 de	39.63 ± 3.20 ab	41.04 ± 3.49 a	23.91 ± 4.31 de	29.52 ± 4.46 bcd	37.85 ± 0.93 abc	28.39 ± 8.15 cde	17.94 ± 6.22 e
Basidiomycota	24.66 ± 0.45 cd	21.33 ± 2.00 de	18.79 ± 5.49 de	14.11 ± 8.82 e	32.33 ± 14.81 bc	22.18 ± 1.51 de	18.02 ± 1.32 de	40.80 ± 5.45 ab	21.67 ± 2.23 de	23.20 ± 0.61 cde	44.06 ± 4.29 a	17.71 ± 7.75 de
Zygomycota	17.89 ± 2.59 b	15.01 ± 2.33 b	12.24 ± 5.06 b	39.88 ± 20.38 a	26.85 ± 20.30 ab	11.89 ± 5.64 b	19.25 ± 2.82 b	11.12 ± 1.74 b	21.72 ± 7.94 b	10.41 ± 1.72 b	9.60 2.93 b	19.26 ± 8.45 b

1, 2 and 4 indicate intercropping with 0, 1 and 3 species of aromatic plants, respectively, to facilitate regression analysis. T model, intercropping with aromatic plants in the clean tillage soil; G model, intercropping with aromatic plants in the natural grass soil. BGS, branch growth stage; FDS, fruit development stage.
