# Supplementary material for: Plant Interaction Patterns Shape the Soil Microbial Community and Nutrient Cycling in Different Intercropping Scenarios of Aromatic Plant Species
Source: Front Microbiol. 2022 May 27;13:888789. doi: 10.3389/fmicb.2022.888789 (PMC9197114; doi:10.3389/fmicb.2022.888789)
Supplement: Supplementary Table S3 — Network properties of soil microbial communities in different pattern. [file Data_Sheet_3.PDF]

Table S3 | Network properties of soil microbial communities in different pattern.

Treatment	T model			G model		
	1	2	4	1	2	4
Nodes (N)	44	52	22	46	76	80
Edges (E)	39	39	19	60	667	555
Positive edges (PE)	32	28	17	41	298	283
Negative edges (NE)	7	11	2	5	369	272
Average degree (AD)	1.773	1.500	1.727	2.609	17.816	13.875
Network diameter (ND)	7	3	3	5	7	10
Average path length (APL)	2.833	1.615	1.471	1.801	2.189	3.016
Average clustering coefficient (ACC)	0.000	0.000	0.000	0.000	0.620	0.582
Modularity (MD)	0.808	0.893	0.748	0.764	0.244	0.310
Density (D)	0.041	0.029	0.082	0.058	0.238	0.176
Number of communities (NC)	9	14	6	9	2	1

1, 2 and 4 indicate intercropping with 0, 1 and 3 species of aromatic plants, respectively, to facilitate regression analysis. T model, intercropping with aromatic plants in the clean tillage soil; G model, intercropping with aromatic plants in the natural grass soil.
