# Supplementary material for: Plant Interaction Patterns Shape the Soil Microbial Community and Nutrient Cycling in Different Intercropping Scenarios of Aromatic Plant Species
Source: Front Microbiol. 2022 May 27;13:888789. doi: 10.3389/fmicb.2022.888789 (PMC9197114; doi:10.3389/fmicb.2022.888789)
Supplement: Supplementary Table S4 — Network properties of soil microbial communities according to development stage. [file Data_Sheet_4.PDF]

Table S4 | Network properties of soil microbial communities according to development stage.

Treatment	BGS			FDS		
	1	2	4	1	2	4
Nodes (N)	42	56	58	14	56	61
Edges (E)	35	106	97	9	83	113
Positive edges (PE)	22	63	57	4	40	67
Negative edges (NE)	20	43	40	5	43	46
Average degree (AD)	1.667	3.786	3.345	1.286	2.964	3.705
Network diameter (ND)	5	9	8	2	13	7
Average path length (APL)	1.893	3.449	3.102	1.438	5.911	2.550
Average clustering coefficient (ACC)	0.000	0.443	0.489	0.000	0.356	0.554
Modularity (MD)	0.810	0.493	0.563	0.716	0.678	0.488
Density (D)	0.041	0.069	0.059	0.099	0.054	0.062
Number of communities (NC)	12	6	9	5	6	10

1, 2 and 4 indicate intercropping with 0, 1 and 3 species aromatic plants, respectively to facilitate regression analysis. BGS, branch growth stage; FDS, fruit development stage.
